# Menstrual cycle features in mothers and daughters in the Avon Longitudinal Study of Parents and Children (ALSPAC)

**DOI:** 10.12688/wellcomeopenres.19774.3

**Published:** 2023-11-24

**Authors:** Gemma Sawyer, Laura D. Howe, Abigail Fraser, Gemma Clayton, Deborah A. Lawlor, Gemma C. Sharp

**Affiliations:** 1 MRC Integrative Epidemiology Unit, University of Bristol, Bristol, England, UK; 2 Population Health Sciences, Bristol Medical School, University of Bristol, Bristol, England, UK; 3 School of Psychology, Faculty of Health and Life Sciences, University of Exeter, Exeter, England, UK

**Keywords:** ALSPAC, menstruation, menstrual cycle, cohort, longitudinal

## Abstract

Problematic menstrual cycle features, including irregular periods, severe pain, heavy bleeding, absence of periods, frequent or infrequent cycles, and premenstrual symptoms, are experienced by high proportions of females and can have substantial impacts on their health and well-being. However, research aimed at identifying causes and risk factors associated with such menstrual cycle features is sparse and limited. This data note describes prospective, longitudinal data collected in a UK birth cohort, the Avon Longitudinal Study of Parents and Children (ALSPAC), on menstrual cycle features, which can be utilised to address the research gaps in this area. Data were collected across 21 timepoints (between the average age of 28.6 and 57.7 years) in mothers (G0) and 20 timepoints (between the average age of 8 and 24 years) in index daughters (G1) between 1991 and 2020. This data note details all available variables, proposes methods to derive comparable variables across data collection timepoints, and discusses important limitations specific to each menstrual cycle feature. Also, the data note identifies broader issues for researchers to consider when utilising the menstrual cycle feature data, such as hormonal contraception, pregnancy, breastfeeding, and menopause, as well as missing data and misclassification.

## Introduction

Problematic menstrual cycle features have been reported to affect high proportions of adolescent girls, women, and people who menstruate. Previous research had indicated that 11–46% of females experience irregular periods ^1– 6^, 42–95% dysmenorrhea (menstrual pain) ^1– 4, 7^, 3–13% amenorrhea (absence of periods) ^5, 8, 9^, 37–78% premenstrual syndrome (PMS) ^1, 4^, 1–19% frequent or infrequent cycles (less than 24 or more than 38 days) ^1, 8, 10^, and 4–58% heavy menstrual bleeding (HMB) ^4, 8, 11^. Whilst most of these estimates come from research conducted in high income countries (HICs), the research conducted in low and middle income countries (LMICs) indicates similar prevalence ranges. However, the estimates do vary considerably, possible due to factors such as age, the setting and population under study, measurement and definition of the feature, and contextual attitudes towards menstruation. Such features may be related to other conditions, such as endometriosis, polycystic ovary syndrome (PCOS), or fibroids; however, many are idiopathic ^10, 12, 13^. These relatively common problematic menstrual cycle features have been associated with several adverse physical and mental health outcomes, including infertility, anaemia, pain sensitivity, sleep disturbances, anxiety, and depression ^7, 8, 14^. Research has also demonstrated a substantial impact on social wellbeing; negatively impacting school, work, relationships, exercise, and health-related quality of life ^8, 15^.

Despite this, there has been relatively limited research to understand the causes and risk factors associated with problematic menstrual cycle features. Previous research has highlighted ethnicity ^16^, family history ^1, 2, 7, 12^, smoking ^6, 12^, high or low BMI ^2, 6, 7^, earlier age at menarche ^2, 6, 7, 12^, presence of other menstrual problems ^1, 7, 12^, and low socioeconomic position ^1, 2, 6, 14, 16, 17^ (SEP) as possible risk factors. However, much of the evidence comes from cross-sectional studies and research in clinical populations or university students. This means that many findings may be unrepresentative of broader populations, reports of menstrual cycle features may be hindered by recall bias, and there is a limited understanding of whether associations reflect causal effects or otherwise (e.g., confounding) and the direction of any causal effect. Further research is therefore necessary to understand the burden, life course risk factors, causes, and consequences of problematic menstrual cycle features, and provide insights into the causes and consequences of within and between woman variation in menstrual features. This would require greater use of prospective, longitudinal data on the menstrual cycle as well as factors that may be confounders, mediators, or moderators to enhance the accuracy of reporting and enable exploration of causal relationships.

Whilst there are limited resources that provide such rich menstrual cycle data, the Avon Longitudinal Study of Parents and Children (ALSPAC) is a longitudinal birth cohort with repeated measures data on features of the menstrual cycle across two generations of females, as well as data on a wide range of physical, psychological, social, and genetic factors. This data note aims to promote the use of the detailed menstrual cycle data available in ALSPAC to address these research needs. We describe the menstrual cycle data collected in the mothers (G0) and index daughters (G1) enrolled in ALSPAC, including repeated measures on menstrual cycle features throughout puberty and into early adulthood from questionnaire and clinic assessments.

## Methods

### ALSPAC

ALSPAC is a longitudinal birth cohort where pregnant women resident in Avon, UK with expected dates of delivery between 1 ^st^ April 1991 and 31 ^st^ December 1992 were invited to take part in the study. The initial number of pregnancies enrolled was 14,541, resulting in 13,988 children who were alive at 1 year of age.

When the oldest children were approximately 7 years of age, an attempt was made to bolster the initial sample with eligible cases who had failed to join the study originally. The total sample size for analyses using any data collected after the age of 7 is therefore 15,447 pregnancies and, of these, 14,901 children were alive at 1 year of age. There were 14,203 unique mothers initially enrolled in study but as a result of the additional phases of recruitment, 14,833 unique mothers enrolled in ALSPAC as of September 2021.

Study data gathered from participants at 22 years and onwards were collected and managed using Research Electronic Data Capture (REDCap) tools hosted at the University of Bristol ^18^. REDCap is a secure, web-based software platform designed to support data capture for research studies.

Further details on ALSPAC have been published elsewhere ^19– 21^. Please note that the study website contains details of all the data that is available through a fully searchable data dictionary and variable search tool ( http://www.bristol.ac.uk/alspac/researchers/our-data/). Ethical approval for the study was obtained from the ALSPAC Ethics and Law Committee and the Local Research Ethics Committees (ALEC; IRB00003312). Detailed information can be found on the study website http://www.bristol.ac.uk/alspac/researchers/research-ethics/. Implicit consent for the use of data collected via questionnaires and clinics was obtained from participants following the recommendations of the ALSPAC Ethics and Law Committee at the time.

**Figure 1.**
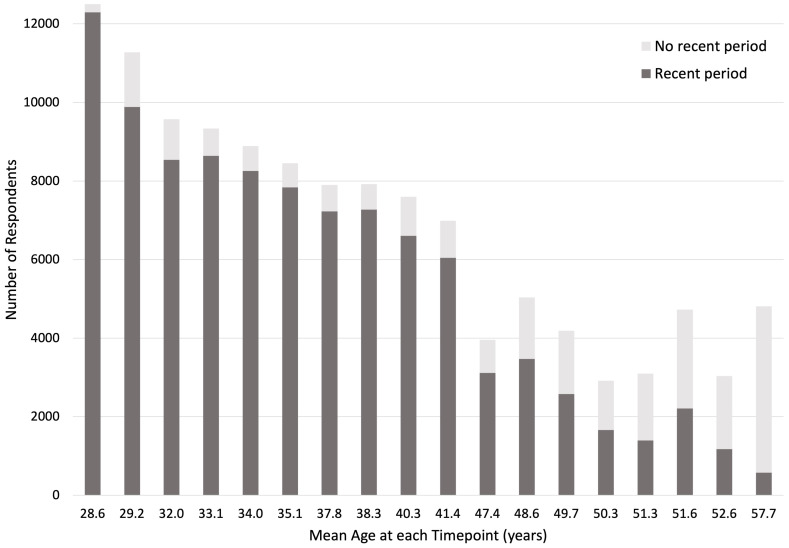
Number of G0 participants (mothers) with recent periods. ‘No recent period’ includes participants who reported no period in the last 12 months, no ‘recent’ periods, or no ‘periods nowadays’.

**Figure 2.**
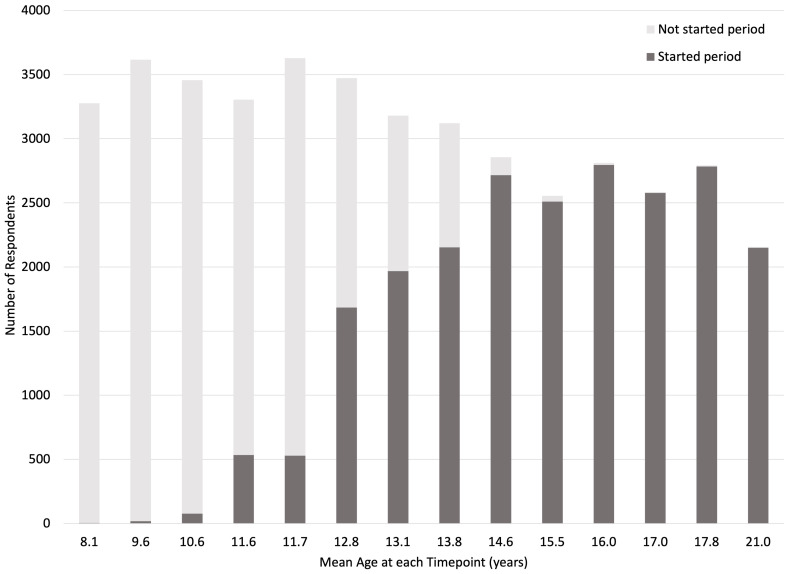
Number of G1 participants (daughters) who have started periods. ‘Started period’ includes participants who responded yes when asked whether they had started their period yet or not at each timepoint.

This data note focuses on two samples: G0 (mothers) and G1 (daughters) who have been asked about several menstrual cycle features at multiple timepoints. The G0 sample comprises mothers who were asked to report on their menstrual cycle features from the index pregnancy to menopause. Figure 1 shows the number of G0 participants responding to questions about periods at each relevant timepoint, and whether they had experienced a recent period. The female offspring (G1) sample comprises female participants who provided information, either reported by their mother or themselves, about menstrual cycle features from age 8 to age 24. Figure 2 shows the number of respondents who had started their period up until age 21.

### Mothers (G0) Data

Participants answered questions relating to menstrual cycle length, absence of periods, regularity, heaviness, pain, and PMS-related symptoms across 17 questionnaires from the index pregnancy (reporting on menstruation prior to pregnancy) up to 28 years following birth of the index child (mean age 28.6 to 57.7 years). Participants also provided data on menstrual cycle features at four clinic assessments from mean age 47.4 to 52.6 years ( Table 1). Table 1 provides an overview of the ALSPAC questionnaires and clinics, participants mean age, and the relevant menstrual cycle feature variables at each timepoint, as well as contraception variables at each available timepoint.

**Table 1.** Source of G0 menstrual cycle and contraception data.

ALSPAC Source File Mean (SD) Age	G0 variable names						
	Cycle length	Amenorrhea	Cycle regularity	Heavy/ prolonged bleeding	Pain	PMS	Contraception
**d Quest** 28.6 (4.86) years *	d012	d012	d011	-	-	-	
**f Quest** 29.2 (4.78) years	-	-	-	-	-	f036 f036a	f068 f068a
**g Quest** 30.0 (4.74) years	-	-	-	-	-	g036 g036a	g054 g054a g133-g141
**h Quest** 32.0 (4.74) years	-	-	h112	h110 h113 h115a	h111 h115b	h028 h028a	h044 h044a h098a-h098h h098j
**j Quest** 33.1 (4.69) years	-	-	j143	j141 j144	j142	j027 j027a	j049 j049a j113- j121
**k Quest** 34.0 (4.67) years	-	-	k1292	k1290 k1293 k1301	k1291 k1302	k1031 k1035	k1051 k1203-k1213
**l Quest** 35.1 (4.62) years	-	-	l3352	l3350 l3353	l3351	l3031 l3360 l3370 l3380 l3390 l3400	l3051 l3300-l3310
**m Quest** 36.1 (4.61) years	-	-	-	m4201	m4202	-	-
**n Quest** 37.8 (5.48) years	n1122	n1122 n1123	n1121	-	-	-	n1130 n1133
**p Quest** 38.3 (4.60) years	-	-	p1262	p1260 p1263	p1261	p1031 p1270- p1274 p1280- p1284	p1061 p1243- p1253
**q Quest** 39.3 (4.61) years	-	-	-	-	q4020	-	q4280
**r Quest** 40.3 (4.63) years	r2082	r2082 r2083	r2081	-	-	-	r2090 r2093 r2173- r2183
**s Quest** 41.4 (4.55) years	-	-	s1262	s1260 s1263	s1261 s4020	s1031 s1270- s1274 s1280- s1284	s1243-s1253 s4280
**FOM1 Clinic** 47.4 (4.52) years		fm1ob120 fm1ob121 fm1ob126	fm1ob130	-	-	-	fm1ob100 fm1ob101
**t Quest** 48.6 (4.49) years	-	t4800 t4801 t4802 t4803 t4804 t4805	t4837	t4835 t4838 t4901	t4836 t4902	t4850-t4859	t4520-t4531 t5414
**u Quest** 49.7 (4.52) years	u1050	-	u1050	-	-	-	u1030-u1034
**FOM2 Clinic** 50.3 (4.42) years		fm2ob120 fm2ob121 fm2ob126	fm2ob130	-	-	-	fm2ob100 fm2ob101
**v Quest** 51.3 (4.49) years	V4831	V4800 V4801 V4802 V4803 V4804 V4805 V4810	V4831 V4837	V4835 V4838 V4901	V4836 V4902	V4840 V4850- V4859	V4550-V4565
**FOM3 Clinic** 51.6 (4.47) years		fm3ob120 fm3ob121 fm3ob126	fm3ob130	-	-	-	fm3ob100 fm3ob101
**FOM4 Clinic** 52.6 (4.42) years		fm4ob120 fm4ob121 fm4ob126	fm4ob130	-	-	-	fm4ob100 fm4ob101
**Y Quest** 57.7 (4.48) years	-	Y5070 Y5080 Y5081 Y5082 Y5083 Y5084 Y5085 Y5100	-	-	-	-	Y5000-Y5014

*12 weeks’ gestation

***Cycle length***

In the first three questionnaires asking about cycle length (mean age 28.6, 37.8, and 40.3 years), participants were asked how many days there were from the start of one period to the start of the next one if their periods were regular. At the mean age 49.7 years questionnaire, participants were asked if their periods were regular and could select yes, alongside multiple options for the length of their cycle, or no. Similarly, at the aged 51.3 years questionnaire, participants were asked how many days there usually were between the start of one period and the start of the next and were provided with multiple categorical options, as well as an option to report that their periods were too irregular to estimate. These questions and variables are summarised in Table 2.

**Table 2.** Original and derived G0 cycle length variables (N (%)).

Question	28.6 years	37.8 years	40.3 years	49.7 years	51.3 years
Original variables					
**If regular, how many days were there from the start of one period to the start of the next one?**					
*Mean (SD)*	27.54 (3.85)	25.90 (6.36)	25.73 (6.57)	-	-
< 15 days *	107 (1.16)	419 (7.24)	388 (7.46)	-	-
15–20 days *	50 (0.54)	47 (0.81)	45 (0.87)	-	-
21–25 days *	1218 (13.15)	873 (15.08)	831 (15.98)	-	-
26–30 days *	7072 (76.35)	4103 (70.89)	3689 (70.96)	-	-
31–35 days *	747 (8.07)	322 (5.56)	226 (4.35)	-	-
36–40 days *	42 (0.45)	16 (0.28)	9 (0.17)	-	-
> 40 days *	26 (0.28)	8 (0.14)	11 (0.21)	-	-
**Are your periods regular?**					
*Yes, every 23 days or less*	-	-	-	283 (10.98)	-
*Yes, between 24 and 35 days*	-	-	-	1420 (55.10)	-
*Yes, more than every 35 days*	-	-	-	86 (3.34)	-
*No*	-	-	-	788 (30.58)	-
**In the years before your last period, how many days do you usually have between the start of one period and** **the start of the next period?**					
*Less than 21 days*	-	-	-	-	329 (11.14)
*21–25 days*	-	-	-	-	594 (20.12)
*26–31 days*	-	-	-	-	1066 (36.11)
*32–39 days*	-	-	-	-	161 (5.45)
*40–50 days*	-	-	-	-	78 (2.64)
*More than 50 days*	-	-	-	-	96 (3.25)
*Too irregular to estimate*	-	-	-	-	628 (21.27)
Derived variables					
*Normal (24–38 days)*	8246 (89.03)	4846 (83.72)	4331 (83.30)	-	-
*Frequent (<24 days)*	978 (10.56)	926 (16.00)	852 (16.39)	-	-
*Infrequent (>38 days)*	38 (0.41)	16 (0.28)	16 (0.31)	-	-

*Continuous responses recoded.

***Amenorrhea***

At the mean age 37.8- and 40.3-year questionnaires, participants who reported not having periods were asked whether this was because of pregnancy, hysterectomy, menopause, or another reason. At the aged 47.4 questionnaire, participants were asked whether they had a period in the last 12 months. Participants were also asked whether they had a period in the last 12 months at the mean aged 51.3 and 57.7 year questionnaires and all of the clinics, as well as additionally being asked whether they had a period in the last 3 months. Those who reported not having a period were subsequently asked to report the reason for this. Responses included surgery, ablation or resection, chemotherapy or radiation therapy, pregnancy or breastfeeding, menopause, various methods of contraception, and other reasons. Table 3 provides a summary of these questions and responses. Moreover, as mentioned above, participants were asked to report the number of days from the start of one period to the start of the next in three questionnaires (mean age 28.6, 37.8, and 40.3 years). These variables may also be informative regarding amenorrhea if participants have reported long cycle lengths (e.g., 84 days or 3 months between two periods).

**Table 3.** Original and derived G0 amenorrhea variables (N (%)).

Question	28.6 years	37.8 years	40.3 years	47.4 years clinic	48.6 years	50.3 years clinic	51.3 years	51.6 years clinic	52.6 years clinic	57.7 years
Original variables										
**If regular, how many days were there from the start of one period to the start of the next one?**										
*Mean (SD)*	27.54 (3.85)	25.90 (6.36)	25.73 (6.57)	-	-	-	-	-	-	-
< 15 days*	107 (1.16)	419 (7.24)	388 (7.46)	-	-	-	-	-	-	-
15–20 days*	50 (0.54)	47 (0.81)	45 (0.87)	-	-	-	-	-	-	-
21–25 days*	1218 (13.15)	873 (15.08)	831 (15.98)	-	-	-	-	-	-	-
26–30 days*	7072 (76.35)	4103 (70.89)	3689 (70.96)	-	-	-	-	-	-	-
31–35 days*	747 (8.07)	322 (5.56)	226 (4.35)	-	-	-	-	-	-	-
36–40 days*	42 (0.45)	16 (0.28)	9 (0.17)	-	-	-	-	-	-	-
> 40 days*	26 (0.28)	8 (0.14)	11 (0.21)	-	-	-	-	-	-	-
**If you have no periods now, is this because of:**										
*Pregnant*	-	92 (12.37)	49 (4.79)	-	-	-	-	-	-	-
*Hysterectomy*	-	183 (24.60)	282 (27.54)	-	-	-	-	-	-	-
*Menopausal*	-	79 (10.62)	213 (20.80)	-	-	-	-	-	-	-
*Other reason*	-	363 (48.79)	467 (45.61)	-	-	-	-	-	-	-
*Don’t know*	-	27 (3.63)	13 (1.27)	-	-	-	-	-	-	-
**In the last 12 months, have you had a period or menstrual bleeding?**										
*Yes*	-	-	-	3470 (69.15)	2669 (64.13)	1671 (56.26)	2206 (46.69)	1454 (46.57)	1175 (38.73)	571 (11.87)
*No*	-	-	-	1548 (30.85)	1493 (35.87)	1299 (43.74)	2519 (53.31)	1668 (53.43)	1859 (61.27)	4239 (88.13)
**In the last 3 months, have you had a period or menstrual bleeding?**										
*Yes*	-	-	-	3149 (90.93)	-	1398 (64.48)	1855 (83.75)	1209 (49.57)	971 (82.64)	406 (71.86)
*No*	-	-	-	314 (9.07)	-	770 (35.52)	360 (16.25)	1230 (50.43)	204 (17.36)	159 (28.14)
**Reason why periods stopped**										
*Hysterectomy*	-	-	-	339 (21.68)	-	199 (15.81)	-	219 (12.81)	233 (12.53)	-
*Ablation or* *resection*	-	-	-	24 (1.53)	-	-	-	-	-	-
*Chemotherapy or* *radiation therapy*	-	-	-	38 (2.43)	-	32 (2.54)	-	43 (2.51)	36 (1.94)	-
*Menopause*	-	-	-	763 (48.79)	-	751 (59.65)	-	1081 (63.22)	1237 (66.54)	-
*Contraceptive:* *hormonal coil*	-	-	-	256 (16.37)	-	-	-	243 (14.21)	203 (10.92)	-
*Contraceptive:* *injection*	-	-	-	62 (3.96)	-	-	-	-	-	-
*Contraceptive:* *implant*	-	-	-	5 (0.32)	-	-	-	-	-	-
*Contraceptive: pill*	-	-	-	59 (3.77)	-	-	-	-	-	-
*Contraceptive: other*	-	-	-	6 (0.38)		-		-	-	
*Other medical* *reason*	-	-	-	12 (0.77)	-	277 (22.00)	-	124 (7.25)	150 (8.07)	-
**Were your periods stopped by:**										
*Surgery*	-	-	-	-	273 (20.15)	-	-	-	-	-
Chemotherapy or radiation therapy	-	-	-	-	48 (3.54)	-	-	-	-	-
Pregnancy or breastfeeding	-	-	-	-	6 (0.44)	-	-	-	-	-
No obvious reason / menopause	-	-	-	-	791 (58.38)	-	-	-	-	-
Other reason	-	-	-	-	237 (17.49)	-	-	-	-	-
**Were your periods stopped by:**										
*Surgery*	-	-	-	-	-	-	325 (14.39)	-	-	-
Chemotherapy or radiation therapy	-	-	-	-	-	-	63 (2.79)	-	-	-
Pregnancy or breastfeeding	-	-	-	-	-	-	–	-	-	-
No obvious reason / menopause	-	-	-	-	-	-	1494 (66.16)	-	-	-
Contraception	-	-	-	-	-	-	372 (16.47)	-	-	-
**Were your periods stopped by:**										
*Surgery*	-	-	-	-	-	-	-	-	-	410 (10.30)
*Chemotherapy or* *radiation therapy*	-	-	-	-	-	-	-	-	-	66 (1.66)
Pregnancy or breastfeeding	-	-	-	-	-	-	-	-	-	<5
*Menopause*	-	-	-	-	-	-	-	-	-	3134 (78.76)
*Contraception*	-	-	-	-	-	-	-	-	-	257 (6.46)
*Other reason*	-	-	-	-	-	-	-	-	-	109 (2.74)
Derived variables										
*Period in last 3* *months*	9262 (NA)	5788 (NA)	5198 (NA)	3149 (62.84)	2669 (64.13)	1398 (47.93)	1855 (39.18)	1209 (39.01)	971 (32.00)	406 (8.45)
*No period in last 3* *months*	<5	<5	<5	1862 (37.16)	1493 (35.87)	1519 (52.07)	2879 (60.82)	1890 (60.99)	2063 (68.00)	4398 (91.55)

Cell counts below 5 have been replaced with <5, and this may include 0.

***Cycle regularity***

Participants were asked whether their periods were regular or not in three of the questionnaires (mean age 28.6, 37.8, and 40.3 years) and were also asked whether their periods were very, moderately, mildly, or not at all irregular in eight of the questionnaires (mean age 32.0, 33.1, 34.0, 35.1, 38.3, 41.4, 48.6, and 51.3 years). In the four clinics (mean age 47.4, 50.3, 51.6, and 52.6 years) and mean age 49.7 years questionnaire, participants were asked about the regularity or length of their menstrual cycle and were able to report that their cycle was not regular or too irregular to estimate their cycle length. Table 4 gives an overview of these variables.

**Table 4.** Original and derived G0 cycle regularity variables (N (%)).

Question	28.6 years	32.0 years	33.1 years	34.0 years	35.1 years	37.8 years	38.3 years	40.3 years	41.4 years	47.4 years clinic	48.6 years	49.7 years	50.3 years clinic	51.3 years	51.6 years clinic	52.6 years clinic
Original variables																
**In the year before this pregnancy, would you say your periods were regular?**																
*Yes*	9880 (80.36)	-	-	-	-	-	-	-	-	-	-	-	-	-	-	-
*No*	2415 (19.64)	-	-	-	-	-	-	-	-	-	-	-	-	-	-	-
**How would you describe your recent periods: Irregular?**																
*Very*	-	737 (8.63)	732 (8.49)	-	665 (8.79)	-	-	-	-	-	-	-	-	-	-	-
*Moderately*	-	670 (7.85)	708 (8.21)	-	721 (9.53)	-	-	-	-	-	-	-	-	-	-	-
*Mildly*	-	1315 (15.40)	1404 (16.28)	-	1319 (17.43)	-	-	-	-	-	-	-	-	-	-	-
*Not at all*	-	5815 (68.12)	5782 (67.03)	-	4862 (64.25)	-	-	-	-	-	-	-	-	-	-	-
**Are your periods irregular?**																
*Very*	-	-	-	931 (11.40)	-	-	872 (12.09)	-	770 (12.87)	-	671 (22.28)	-	-	1115 (26.20)	-	-
*Moderately*	-	-	-	778 (9.52)	-	-	790 (10.95)	-	706 (11.80)	-	454 (15.07)	-	-	857 (20.14)	-	-
*Mildly*	-	-	-	1376 (16.84)	-	-	1254 (17.39)	-	1153 (19.27)	-	570 (18.92)	-	-	823 (19.34)	-	-
*Not at all*	-	-	-	5084 (62.24)	-	-	4297 (59.57)	-	3355 (56.07)	-	1317 (43.73)	-	-	1461 (34.33)	-	-
**Would you say your periods are regular nowadays?**																
*Yes*	-	-	-	-	-	6019 (83.31)	-	5393 (81.65)	-	-	-	-	-	-	-	-
*No*	-	-	-	-	-	1206 (16.69)	-	1212 (18.35)	-	-	-	-	-	-	-	-
**Periods are regular**																
*Yes, every 28–30* *days*	-	-	-	-	-	-	-	-	-	1793 (51.78)	-	-	626 (29.81)	-	489 (23.68)	352 (29.98)
*Yes, < every 28* *days*	-	-	-	-	-	-	-	-	-	343 (9.90)	-	-	198 (9.43)	-	140 (6.78)	102 (8.69)
*Yes, > every 30* *days*	-	-	-	-	-	-	-	-	-	111 (3.21)	-	-	68 (3.24)	-	70 (3.39)	32 (2.73)
*No*	-	-	-	-	-	-	-	-	-	1216 (35.11)	-	-	1208 (57.52)	-	1366 (66.15)	688 (58.60)
**Are your periods regular?**																
*Yes, every 23* *days or less*	-	-	-	-	-	-	-	-	-	-	-	283 (10.98)	-	-	-	-
*Yes, between 24* *and 35 days*	-	-	-	-	-	-	-	-	-	-	-	1420 (55.10)	-	-	-	-
*Yes, more than* *every 35 days*	-	-	-	-	-	-	-	-	-	-	-	86 (3.34)	-	-	-	-
*No*	-	-	-	-	-	-	-	-	-	-	-	788 (30.58)	-	-	-	-
**In the years before your last period, how many days do you usually have between the start of one period and the start of the next period?**																
*Less than 21* *days*	-	-	-	-	-	-	-	-	-	-	-	-	-	329 (11.14)	-	-
*21–25 days*	-	-	-	-	-	-	-	-	-	-	-	-	-	594 (20.12)	-	-
*26–31 days*	-	-	-	-	-	-	-	-	-	-	-	-	-	1066 (36.11)	-	-
*32–39 days*	-	-	-	-	-	-	-	-	-	-	-	-	-	161 (5.45)	-	-
*40–50 days*	-	-	-	-	-	-	-	-	-	-	-	-	-	78 (2.64)	-	-
*More than 50* *days*	-	-	-	-	-	-	-	-	-	-	-	-	-	96 (3.25)	-	-
*Too irregular to* *estimate*	-	-	-	-	-	-	-	-	-	-	-	-	-	628 (21.27)	-	-
Derived variables																
*Regular*	9880 (80.36)	7130 (83.52)	7186 (83.31)	6460 (79.08)	6181 (81.68)	6019 (83.31)	5551 (76.96)	5393 (81.65)	4508 (75.33)	2247 (64.89)	1887 (62.65)	1789 (69.42)	892 (42.48)	2284 (53.67)	699 (33.85)	486 (41.40)
*Irregular*	2415 (19.64)	1407 (16.48)	1440 (16.69)	1709 (20.92)	1386 (18.32)	1206 (16.69)	1662 (23.04	1212 (18.35)	1476 (24.67)	1216 (35.11)	1125 (37.35)	788 (30.58)	1208 (57.52)	1972 (46.33)	1366 (66.15)	688 (58.60)

***Heavy or prolonged bleeding***

G0 participants were asked about heavy bleeding and prolonged bleeding separately. Participants were asked whether their periods were very, moderately, mildly, or not at all heavy in eight questionnaires (mean age 32.0, 33.1, 34.0, 35.1, 38.3, 41.4, 48.6, and 51.3 years). In addition, participants were asked whether they had had a dilation and curettage (D&C)/scrape due to heavy bleeding in five questionnaires (mean age 32.0, 34.0, 36.1, 48.6, and 51.3 years). In terms of prolonged bleeding, participants were asked to report the number of days that bleeding usually lasts for in eight questionnaires (mean age 32.0, 33.1, 34.0, 35.1, 38.3, 41.4, 48.6, and 51.3 years). These questions are summarised in Table 5.

**Table 5.** Original and derived G0 heavy and/or prolonged bleeding variables (N (%)).

Question	32.0 years	33.1 years	34.0 years	35.1 years	36.1 years	38.3 years	41.4 years	48.6 years	51.3 years
Original variables									
**How heavy are your periods?**									
*Very*	1321 (15.47)	1412 (16.35)	1408 (17.05)	1448 (18.48)	-	1305 (17.95)	1127 (18.65)	765 (24.56)	1293 (29.81)
*Moderately*	4430 (51.89)	4522 (52.35)	4342 (52.59)	4049 (51.68)	-	3712 (51.06)	2961 (48.99)	1293 (41.51)	1537 (35.44)
*Mildly*	2001 (23.44)	1884 (21.81)	1810 (21.92)	1775 (22.66)	-	1583 (21.77)	1361 (22.52)	710 (22.79)	925 (21.33)
*Not at all*	785 (9.20)	820 (9.49)	696 (8.43)	562 (7.17)	-	670 (9.22)	595 (9.84)	347 (11.14)	582 (13.42)
**How many days does bleeding usually last?**									
*Mean (SD)*	5.26 (2.01)	5.26 (1.89)	5.23 (1.86)	5.22 (1.85)	-	5.17 (1.85)	5.19 (2.14)	5.31 (2.35)	5.77 (3.14)
< 3 days *	129 (1.52)	157 (1.85)	165 (20.04)	143 (1.85)	-	188 (2.72)	191 (3.33)	132 (4.36)	139 (3.34)
*3 days*	654 (7.72)	673 (7.95)	655 (8.12)	705 (9.13)	-	65 (9.43)	564 (9.83)	318 (10.50)	370 (8.89)
*4 days*	1793 (21.16)	1732 (20.46)	1708 (21.16)	1601 (20.74)	-	1455 (21.08)	1176 (20.50)	563 (18.59)	645 (15.50)
*5 days*	3188 (37.62)	3073 (36.30)	2900 (35.93)	2737 (35.46)	-	2376 (34.42)	1979 (34.50)	962 (31.76)	1338 (32.16)
*6 days*	1276 (15.06)	1331 (15.72)	1227 (15.20)	1174 (15.21)	-	976 (14.14)	796 (13.88)	402 (13.27)	560 (13.46)
*7 days*	997 (11.77)	1055 (12.46)	972 (12.04)	942 (12.20)	-	909 (13.17)	698 (12.17)	433 (14.30)	661 (15.89)
*8 days*	179 (2.11)	182 (2.15)	181 (2.24)	181 (2.34)	-	139 (2.01)	124 (2.16)	65 (2.15)	106 (2.55)
*9 days*	55 (0.65)	63 (0.74)	63 (0.78)	58 (0.75)	-	51 (0.74)	41 (0.71)	28 (0.92)	48 (1.15)
< 9 days *	203 (2.40)	199 (2.35)	200 (2.48)	178 (2.31)	-	158 (2.29)	167 (2.91)	126 (4.16)	294 (7.07)
**Have you ever had a dilatation and curettage (D&C / scrape)? If yes, was this because of: heavy periods?**									
*Yes*	172 (1.84)	-	62 (28.84)	-	73 (52.52)	-	-	116 (77.33)	146 (74.11)
*No*	9179 (98.16)	-	153 (71.16)	-	66 (47.48)	-	-	34 (22.67)	51 (25.89)
Derived variables									
Binary Heavy Bleeding **									
*Heavy*	5751 (67.37)	5934 (68.70)	5750 (69.65)	5497 (70.17)	-	5017 (69.01)	4088 (67.64)	2058 (66.07)	2830 (65.25)
*Not heavy*	2786 (32.63)	2704 (31.30)	2506 (30.35)	2337 (29.83)	-	2253 (30.99)	1956 (32.36)	1057 (33.93)	1507 (34.75)
**Binary Prolonged Bleeding**									
*More than 8 days*	258 (3.04)	262 (3.10)	263 (3.26)	236 (3.06)	-	209 (3.03)	208 (3.63)	154 (5.08)	342 (8.22)
*8 days or less*	8216 (96.96)	8203 (96.90)	7808 (96.74)	7483 (96.94)	-	6694 (96.97)	5528 (96.37)	2875 (94.92)	3819 (91.78)
**Binary Heavy or Prolonged Bleeding**									
*Heavy or prolonged bleeding*	5780 (68.15)	5961 (69.67)	5781 (70.65)	5527 (71.03)	-	5037 (70.42)	4117 (69.54)	2083 (67.65)	2868 (67.74)
*Neither heavy nor prolonged bleeding*	2701 (31.85)	2595 (30.33)	2402 (29.35)	2254 (28.97)	-	2116 (29.58)	1803 (30.46)	996 (32.35)	1366 (32.26)

*Continuous responses recoded. **Heavy bleeding is defined as reporting ‘very’ or ‘moderately’ heavy periods, whereas not heavy is defined as reporting periods that are ‘mildly’ or ‘not at all’ heavy.

***Menstrual pain***

Participants were asked whether their periods were very, moderately, mildly, or not at all painful in eight questionnaires (mean age 32.0, 33.1, 34.0, 35.1, 38.3, 41.4, 48.6, and 51.3 years) ( Table 6). Also, respondents were asked whether they had had a D&C/scrape due to painful periods in five questionnaires (mean age 32.0, 34.0, 36.1, 48.6, and 51.3 years) and whether they had used any medicines due to painful periods in two questionnaires (mean age 39.3 and 41.4 years).

**Table 6.** Original and derived G0 menstrual pain variables (N (%)).

Question	32.0 years	33.1 years	34.0 years	35.1 years	36.1 years	38.3 years	39.3 years	41.4 years	48.6 years	51.3 years
Original variables										
**How painful are your periods?**										
*Very*	526 (6.16)	592 (6.85)	581 (7.07)	610 (7.85)	-	559 (7.73)	-	459 (7.64)	263 (8.56)	466 (10.86)
*Moderately*	2063 (24.17)	2302 (26.65)	2217 (26.97)	2231 (28.73)	-	2005 (27.72)	-	1662 (27.66)	798 (25.97)	1084 (25.27)
*Mildly*	3388 (39.69)	3205 (37.11)	3256 (39.61)	3017 (38.85)	-	2820 (38.99)	-	2242 (37.32)	1127 (36.67)	1441 (33.59)
*Not at all*	2560 (29.99)	2538 (29.39)	2166 (26.35)	1908 (24.57)	-	1848 (25.55)	-	1645 (27.38)	885 (28.80)	1299 (30.28)
**Have you ever had a dilatation and curettage (D&C / scrape)? If yes, was this because of: painful periods?**										
*Yes*	134 (1.43)	-	48 (23.76)	-	48 (40.00)	-	-	-	52 (52.00)	60 (43.17)
*No*	9217 (98.57)	-	154 (76.24)	-	72 (60.00)	-	-	-	48 (48.00)	79 (56.83)
**Please indicate if you have used any medicines in the last 12 months: painful periods**										
*Yes*	-	-	-	-	-	-	2310 (28.37)	1909 (26.85)	-	-
*No*	-	-	-	-	-	-	5833 (71.63)	5200 (73.15)	-	-
Derived variables										
Painful *	2589 (30.33)	2894 (33.51)	2798 (34.04)	2841 (36.58)	-	2564 (35.45)	-	2121 (35.30)	1061 (34.53)	1550 (36.13)
Not painful *	5948 (69.67)	5743 (66.49)	5422 (65.96)	4925 (63.42)	-	4668 (64.55)	-	3887 (64.70)	2012 (65.47)	2740 (63.87)

*Painful periods are defined as reporting ‘very’ or ‘moderately’ painful periods, whereas not painful is defined as reporting periods that are ‘mildly’ or ‘not at all’ painful.

***Premenstrual symptoms***

In eight questionnaires, participants were asked if they had any recent ‘problems with their period’ (mean age 29.2, 30.0, 32.0, 33.1, 34.0, 35.1, 38.3, and 41.1 years). Additionally, at the mean age 34.0 years questionnaire, participants were asked whether they had ‘pre-menstrual tension’ in the past year, and at the mean age 51.3 years questionnaire, participants were asked whether they had ‘particular problems’ in the days before or during their periods. Furthermore, in the latter five questionnaires (mean age 35.1, 38.3, 41.4, 48.6, and 51.3 years), those who reported having experienced problems with their periods were also asked which symptoms they experienced (very fatigued, irritable, depressed, anxious, or other) and whether each of these symptoms was experienced before their periods, during, or both. Table 7 summarises these questions and variables.

**Table 7.** Original and derived G0 premenstrual symptoms variables (N (%)).

Question	29.2 years	30.0 years	32.0 years	33.1 years	34.0 years	35.1 years	38.3 years	41.4 years	48.6 years	51.3 years
Original variables										
Have you had any problems with your period since your baby was born / toddler was 8 months old / study child was 18 months old / child’s 5th birthday / child’s 6th birthday / child’s 10th birthday?										
*Yes, and saw a doctor*	1135 (10.08)	1092 (10.65)	1148 (11.80)	-	-	1146 (13.39)	1357 (17.09)	984 (14.09)	-	-
*Yes, and did not see a doctor*	980 (8.70)	670 (6.54)	601 (6.18)	-	-	867 (10.13)	684 (8.62)	603 (8.63)	-	-
*No*	9150 (81.23)	8488 (82.81)	7976 (82.02)	-	-	6544 (76.48)	5898 (74.29)	5397 (77.28)	-	-
Have you had any problems with your period in the last year?										
*Yes, and saw a doctor*	-	-	-	1284 (13.39)	-	-	-	-	-	-
*Yes, and did not see a doctor*	-	-	-	752 (7.84)	-	-	-	-	-	-
*No*	-	-	-	7550 (78.76)	-	-	-	-	-	-
In the past year, have you had problems with your period?										
*Yes, and saw a doctor*	-	-	-	-	1128 (12.61)	-	-	-	-	-
*Yes, and did not see a doctor*	-	-	-	-	697 (7.79)	-	-	-	-	-
*No*	-	-	-	-	7119 (79.60)	-	-	-	-	-
In the past year, have you had pre-menstrual tension?										
*Yes, and saw a doctor*	-	-	-	-	428 (4.79)	-	-	-	-	-
*Yes, and did not see a doctor*	-	-	-	-	2970 (33.22)	-	-	-	-	-
*No*	-	-	-	-	5542 (61.99)	-	-	-	-	-
Do you generally find in the days before or during your periods you have particular problems?										
*Yes*	-	-	-	-	-	-	-	-	-	2241 (52.89)
*No*	-	-	-	-	-	-	-	-	-	1996 (47.11)
Do you generally find in the days before or during your periods you have particular problems?										
*Very fatigued before*	-	-	-	-	-	3252 (37.78)	3016 (37.67)	2523 (35.73)	1264 (37.51)	1121 (26.18)
*Very fatigued during*	-	-	-	-	-	2534 (29.47)	2266 (28.36)	1857 (26.35)	1049 (31.13)	768 (17.96)
*Irritable before*	-	-	-	-	-	6350 (73.71)	5826 (72.56)	4683 (66.03)	2107 (62.52)	1744 (40.66)
*Irritable during*	-	-	-	-	-	1954 (22.74)	1479 (18.51)	1142 (16.24)	601 (17.83)	419 (9.80)
*Depressed before*	-	-	-	-	-	2682 (31.19)	2221 (27.79)	1598 (22.67)	864 (25.64)	759 (17.74)
*Depressed during*	-	-	-	-	-	807 (9.40)	642 (8.05)	458 (6.52)	270 (8.01)	234 (5.47)
*Anxious before*	-	-	-	-	-	2038 (23.71)	1662 (20.82)	1380 (19.59)	635 (18.84)	590 (13.79)
*Anxious during*	-	-	-	-	-	564 (6.57)	461 (5.78)	356 (5.07)	193 (5.73)	200 (4.68)
*Other before*	-	-	-	-	-	1149 (13.37)	970 (12.16)	954 (13.57)	371 (11.01)	369 (8.64)
*Other during*	-	-	-	-	-	476 (5.54)	414 (5.19)	388 (5.53)	134 (3.98)	156 (3.65)
Derived variables										
Binary variable: very fatigued *										
*Very fatigued*	-	-	-	-	-	4413 (51.26)	4114 (51.35)	3396 (48.03)	1662 (49.32)	1466 (34.18)
*Not very fatigued*	-	-	-	-	-	4196 (48.74)	3898 (48.65)	3675 (51.97)	1708 (50.68)	2823 (65.82)
Binary variable: irritable *										
*Irritable*	-	-	-	-	-	6679 (77.53)	6098 (75.92)	4903 (69.12)	2183 (64.78)	1860 (43.34)
*Not irritable*	-	-	-	-	-	1936 (22.47)	1934 (24.08)	2190 (30.88)	1187 (35.22)	2432 (56.66)
Binary variable: depressed *										
*Depressed*	-	-	-	-	-	2955 (34.37)	2454 (30.69)	1787 (25.34)	932 (27.66)	866 (20.22)
*Not depressed*	-	-	-	-	-	5643 (65.63)	5541 (69.31)	5264 (74.66)	2438 (72.34)	3416 (79.78)
Binary variable: anxious *										
*Anxious*	-	-	-	-	-	2252 (26.20)	1859 (23.27)	1522 (21.60)	681 (20.21)	695 (16.24)
*Not anxious*	-	-	-	-	-	6345 (73.80)	6129 (76.73)	5524 (78.40)	2689 (79.79)	3585 (83.76)
Binary variable: other symptoms *										
*Other symptoms*	-	-	-	-	-	1302 (15.15)	1120 (14.04)	1074 (15.28)	415 (12.31)	444 (10.39)
*No other symptoms*	-	-	-	-	-	7292 (84.85)	6857 (85.96)	5957 (84.72)	2955 (87.69)	3830 (89.61)
Number of PMS-related symptoms										
*0*	-	-	-	-	-	1502 (17.49)	1467 (18.40)	1689 (24.07)	806 (23.92)	2117 (49.59)
*1*	-	-	-	-	-	1794 (20.88)	1727 (21.67)	1504 (21.43)	767 (22.76)	548 (12.84)
*2*	-	-	-	-	-	2167 (25.23)	2134 (26.77)	1743 (24.84)	831 (24.66)	676 (15.84)
*3*	-	-	-	-	-	1448 (16.86)	1324 (16.61)	1055 (15.03)	507 (15.04)	444 (10.40)
*4*	-	-	-	-	-	1329 (15.47)	1066 (13.37)	810 (11.54)	372 (11.04)	393 (9.21)
*5*	-	-	-	-	-	350 (4.07)	253 (3.17)	217 (3.09)	87 (2.58)	91 (2.13)

*Participants are classified as experiencing the PMS-related symptom (very fatigued, irritable, depressed, anxious, or other) if they reported the symptom either before or during their period, whereas they are classified as not experiencing the symptom if they did not report the symptom before and during their period.

### Daughters (G1) Data

Participants who had started their periods reported on menstrual regularity, heaviness, pain, cycle length, absence of periods, and PMS-related symptoms at multiple timepoints ranging from average 8 to 24 years. This included nine ‘puberty’ questionnaires, where mothers reported on their daughter’s menstrual cycle at age 8.1, 9.6, 10.6, 11.6, and 13.1 years before participants reported on their own menstrual cycle at age 14.6, 15.5, 16, and 17 years. Mothers also responded on behalf of their daughters at three ‘child-based’ questionnaires at age 13.1, 13.8, and 16.5 years. Participants answered further questions regarding their menstrual cycle features at two later ‘child-completed’ questionnaires (age 19.6 and 21 years) and six clinic assessments (age 11.7, 12.8, 13.8, 15.5, 17.8, and 24 years). Table 8 provides an overview of the relevant ALSPAC variables at each timepoint according to menstrual cycle features, as well as contraception variables at each available timepoint.

**Table 8.** Source of G1 menstrual cycle feature and contraception data.

ALSPAC Source File Timepoint	G1 variable names						
	Cycle length	Amenorrhea	Cycle regularity	Heavy/prolonged bleeding	Dysmenorrhea/pain	PMS	Contraception
**pub1 Puberty Quest** 8.1 years	pub117	pub117	-	pub115 pub116 pub120	pub122 pub123	-	pub127
**pub2 Puberty Quest** 9.6 years	pub217	pub217	-	pub215 pub216 pub220	pub222 pub223	-	pub227
**pub3 Puberty Quest** 10.6 years	pub317	pub317	-	pub315 pub316 pub320 pub321	pub322 pub323	-	pub327
**pub4 Puberty Quest** 11.6 years	pub417	pub417	-	pub415 pub416 pub420 pub421	pub422 pub423	-	pub427
**F11 Clinic** 11.7 years	-	-	femn013	-	-	-	-
**tf1 Clinic** 12.8 years	ff2094	ff2094	ff2093	-	-	-	-
**pub5 Puberty Quest** 13.1 years	pub517	pub517	-	pub515 pub516 pub520 pub521	pub522 pub523	-	pub527
**ta Child-Based Quest** 13.1 years	-	ta6190 ta6191	-	-	-	-	-
**tb Child-Based Quest** 13.8 years	-	tb8390 tb8391	-	-	-	-	-
**tf2 Clinic** 13.8 years	-	-	fg6193	-	-	-	-
**pub6 Puberty Quest** 14.6 years	pub617	pub617	-	pub615 pub616 pub620 pub621	pub622 pub623	-	pub627
**pub7 Puberty Quest** 15.5 years	pub717	pub717	-	pub715 pub716 pub720 pub721	pub722 pub723 pub724	-	pub727
**pub8 Puberty Quest** 16 years	pub817	pub817	-	pub815 pub816 pub820 pub821	pub822 pub823	-	pub827
**tc Child-Based Quest** 16.5 years	-	tc5200 tc5201	-	-	-	-	-
**pub9 Puberty Quest** 17 years	pub917	pub917	-	pub915 pub916 pub920 pub921	pub922 pub923	-	pub927
**tf4 Clinic** 17.8 years	-	-	FJMS040	-	-	-	FJMS010-FJMS014
**ccxf Quest** 19.6 years	ccxf3000	-	ccxf3000	-	-	-	ccxf2000-ccxf2004
**YPA Quest** 21 years	YPA7042	YPA7010 YPA7020-YPA7025	YPA7042 YPA7052	YPA7050	YPA7051	YPA7060-YPA7075	YPA3270-YPA3291
**F24 Clinic** 24 years	FKFH1020 FKFH1021	FKFH1020	FKFH1021	-	-	-	FKFH1040

***Cycle length***

In the nine puberty questionnaires, as well as in the 12.8 year clinic, participants (or their mothers up to 13.1 years) were asked to report the length of their menstrual cycle. In two later questionnaires (19.6 and 21 years), participants were asked about their cycle length but were given multiple categorical options instead of reporting the exact number of days. At the age 24 clinic, participants were asked to provide the exact number of days of their usual menstrual cycle or, if they were unsure, they could select an approximate length from multiple categorical options. Table 9 provides an overview of these questions and variables.

**Table 9.** Original and derived G1 cycle length variables (N (%)).

Question	8.1 years	9.6 years	10.6 years	11.6 years	12.8 years clinic	13.1 years	14.6 years	15.5 years	16 years	17 years	19.6 years	21 years	24 years clinic
Original variables													
**In the past year, what was the usual length of your daughter’s menstrual cycle?**													
*Mean (SD)*	NA	18 (18.84)	27.13 (14.86)	26.56 (11.29)	-	26.20 (10.09)	-	-	-	-	-	-	-
< 15 days *	<5	<5	6 (15.00)	38 (12.54)	-	140 (12.96)	-	-	-	-	-	-	-
15–20 days *	<5	<5	<5	8 (2.64)	-	14 (1.30)	-	-	-	-	-	-	-
21–25 days *	<5	<5	6 (15.00)	40 (13.20)	-	125 (11.57)	-	-	-	-	-	-	-
26–30 days *	<5	<5	17 (42.50)	175 (57.76)	-	651 (60.28)	-	-	-	-	-	-	-
31–35 days *	<5	<5	7 (17.50)	22 (7.26)	-	94 (8.70)	-	-	-	-	-	-	-
36–40 days *	<5	<5	<5	6 (1.98)	-	15 (1.39)	-	-	-	-	-	-	-
> 40 days *	<5	<5	<5	14 (4.62)	-	41 (3.80)	-	-	-	-	-	-	-
**If your periods are regular, how long on average would you say your cycle is? (i.e. number of days between each period e.g. 30, 28)**													
*Mean (SD)*	-	-	-	-	28.30 (2.40)	-	-	-	-	-	-	-	-
< 15 days *	-	-	-	-	<5	-	-	-	-	-	-	-	-
15–20 days *	-	-	-	-	<5	-	-	-	-	-	-	-	-
21–25 days *	-	-	-	-	44 (5.44)	-	-	-	-	-	-	-	-
26–30 days *	-	-	-	-	726 (89.74)	-	-	-	-	-	-	-	-
31–35 days *	-	-	-	-	29 (3.58)	-	-	-	-	-	-	-	-
36–40 days *	-	-	-	-	<5	-	-	-	-	-	-	-	-
> 40 days *	-	-	-	-	<5	-	-	-	-	-	-	-	-
**In the past year, how many days were there usually between your periods?**													
*Mean (SD)*	-	-	-	-	-	-	28.35 (7.12)	27.81 (4.97)	27.62 (5.62)	27.32 (5.28)	-	-	-
< 15 days *	-	-	-	-	-	-	13 (1.39)	12 (1.31)	11 (0.91)	11 (0.89)	-	-	-
15–20 days *	-	-	-	-	-	-	18 (1.93)	12 (1.31)	37 (3.08)	14 (1.13)	-	-	-
21–25 days *	-	-	-	-	-	-	138 (14.79)	168 (18.36)	229 (19.04)	311 (25.20)	-	-	-
26–30 days *	-	-	-	-	-	-	636 (68.17)	610 (66.67)	791 (65.75)	773 (62.64)	-	-	-
31–35 days *	-	-	-	-	-	-	92 (9.86)	89 (9.73)	109 (9.06)	98 (7.94)	-	-	-
36–40 days *	-	-	-	-	-	-	12 (1.29)	12 (1.31)	14 (1.16)	16 (1.30)	-	-	-
> 40 days *	-	-	-	-	-	-	24 (2.57)	12 (1.31)	12 (1.00)	11 (0.89)	-	-	-
**Are your periods regular?**													
*Yes, every 23 days or less*	-	-	-	-	-	-	-	-	-	-	423 (22.23)	-	-
*Yes, between 24 and 35 days*	-	-	-	-	-	-	-	-	-	-	973 (51.13)	-	-
*Yes, > every 35 days*	-	-	-	-	-	-	-	-	-	-	47 (2.47)	-	-
*No*	-	-	-	-	-	-	-	-	-	-	460 (24.17)	-	-
**How many days do you usually have between the start of one period and the start of the next period?**													
*Less than 21 days*	-	-	-	-	-	-	-	-	-	-	-	103 (5.03)	-
*21–25 days*	-	-	-	-	-	-	-	-	-	-	-	697 (34.07)	-
*26–31 days*	-	-	-	-	-	-	-	-	-	-	-	752 (36.75)	-
*32–39 days*	-	-	-	-	-	-	-	-	-	-	-	115 (5.62)	-
*40–50 days*	-	-	-	-	-	-	-	-	-	-	-	38 (1.86)	-
*More than 50 days*	-	-	-	-	-	-	-	-	-	-	-	40 (1.96)	-
*Too irregular to estimate*	-	-	-	-	-	-	-	-	-	-	-	301 (14.71)	-
**What is the length of your usual menstrual cycle (the interval from first day of period to first day of next period), when you are NOT using oral contraception, injections, or implant?**													
*Mean (SD)*	-	-	-	-	-	-	-	-	-	-	-	-	25.74 (9.47)
< 15 days *	-	-	-	-	-	-	-	-	-	-	-	-	122 (11.87)
15–20 days *	-	-	-	-	-	-	-	-	-	-	-	-	13 (1.26)
21–25 days *	-	-	-	-	-	-	-	-	-	-	-	-	154 (14.98)
26–30 days *	-	-	-	-	-	-	-	-	-	-	-	-	645 (62.74)
31–35 days *	-	-	-	-	-	-	-	-	-	-	-	-	69 (6.71)
36–40 days *	-	-	-	-	-	-	-	-	-	-	-	-	11 (1.07)
> 40 days *	-	-	-	-	-	-	-	-	-	-	-	-	14 (1.36)
**If you don’t know exactly, would you say it was:**													
*Less than 25 days*	-	-	-	-	-	-	-	-	-	-	-	-	154 (12.10)
*25–34 days*	-	-	-	-	-	-	-	-	-	-	-	-	833 (65.44)
*35–60 days*	-	-	-	-	-	-	-	-	-	-	-	-	72 (5.66)
*More than 60 days*	-	-	-	-	-	-	-	-	-	-	-	-	15 (1.18)
*Too irregular to estimate*	-	-	-	-	-	-	-	-	-	-	-	-	199 (15.63)
Derived variables													
*Normal (24–38 days)*	<5	<5	26 (74.29)	221 (82.46)	776 (95.92)	806 (85.38)	788 (85.47)	762 (83.83)	981 (82.02)	975 (79.40)	-	-	782 (85.56)
*Frequent (<24 days)*	<5	<5	6 (17.14)	31 (11.57)	28 (3.46)	87 (9.22)	104 (11.28)	128 (14.08)	197 (16.47)	232 (18.89)	-	-	112 (12.25)
*Infrequent (>38 days)*	<5	<5	<5	16 (5.97)	5 (0.62)	51 (5.40)	30 (3.25)	19 (2.09)	18 (1.51)	21 (1.71)	-	-	20 (2.19)

Cell counts below 5 have been replaced with <5, and this may include 0. *Continuous values recoded. **Variables derived in line with clinical guidelines. 24–38 days is considered a normal cycle length, whereas less than 24 days is a frequent menstrual cycle and more than 38 days is an infrequent menstrual cycle.

***Amenorrhea***

In the 13.1, 13.8, and 16.5 year child-based questionnaires, mothers were asked whether there had been months when their daughter’s periods had not happened at all (if they had started regular periods) and subsequently, if yes, whether she had a period in the last three months. Similarly, at age 21, participants were asked if they had a period in the last three months and, if not, why their periods had stopped. Options included surgery, chemotherapy or radiation therapy, pregnancy or breastfeeding, no obvious reason or menopause, contraception, or that their periods had not started yet. Table 10 summarises these questions. Also, as discussed above, participants reported the length of their menstrual cycle in the nine puberty questionnaires and the 12.8 and 24 year clinic. These variables could provide further insight into amenorrhea where participants have reported long menstrual cycles (e.g., 84 days or 3 months between two periods).

**Table 10.** Original and derived G1 amenorrhea variables (N (%)).

Question	8.1 years	9.6 years	10.6 years	11.6 years	12.8 years clinic	13.1 years	13.1 years CBQ	13.8 years CBQ	14.6 years	15.5 years	16.0 years	16.5 years CBQ	17.0 years	21.0 years	24.0 years clinic
Original variables															
**In the past year, what was the usual length of your daughter’s menstrual cycle?**															
*Mean (SD)*	NA	18 (18.84)	27.13 (14.86)	26.56 (11.29)	-	26.20 (10.09)	-	-	-	-	-	-	-	-	-
< 15 days *	<5	<5	6 (15.00)	38 (12.54)	-	140 (12.96)	-	-	-	-	-	-	-	-	-
15–20 days *	<5	<5	<5	8 (2.64)	-	14 (1.30)	-	-	-	-	-	-	-	-	-
21–25 days *	<5	<5	6 (15.00)	40 (13.20)	-	125 (11.57)	-	-	-	-	-	-	-	-	-
26–30 days *	<5	<5	17 (42.50)	175 (57.76)	-	651 (60.28)	-	-	-	-	-	-	-	-	-
31–35 days *	<5	<5	7 (17.50)	22 (7.26)	-	94 (8.70)	-	-	-	-	-	-	-	-	-
36–40 days *	<5	<5	<5	6 (1.98)	-	15 (1.39)	-	-	-	-	-	-	-	-	-
> 40 days *	<5	<5	<5	14 (4.62)	-	41 (3.80)	-	-	-	-	-	-	-	-	-
**If your periods are regular, how long on average would you say your cycle is? (i.e. number of days between each period e.g. 30, 28)**															
*Mean (SD)*	-	-	-	-	28.30 (2.40)	-	-	-	-	-	-	-	-	-	-
< 15 days *	-	-	-	-	<5	-	-	-	-	-	-	-	-	-	-
15–20 days *	-	-	-	-	<5	-	-	-	-	-	-	-	-	-	-
21–25 days *	-	-	-	-	44 (5.44)	-	-	-	-	-	-	-	-	-	-
26–30 days *	-	-	-	-	726 (89.74)	-	-	-	-	-	-	-	-	-	-
31–35 days *	-	-	-	-	29 (3.58)	-	-	-	-	-	-	-	-	-	-
36–40 days *	-	-	-	-	<5	-	-	-	-	-	-	-	-	-	-
> 40 days *	-	-	-	-	<5	-	-	-	-	-	-	-	-	-	-
**If she (your daughter) has started her regular periods, have there been any months when the period didn’t happen at all?**															
*Yes*	-	-	-	-	-	-	487 (21.66)	605 (20.88)	-	-	-	429 (14.92)	-	-	-
*No*	-	-	-	-	-	-	1596 (71.00)	2107 (72.71)	-	-	-	2185 (75.97)	-	-	-
*Don’t know*	-	-	-	-	-	-	165 (7.34)	186 (6.42)	-	-	-	262 (9.11)	-	-	-
**If yes, has she had any periods in the last 3 months?**															
*Yes*	-	-	-	-	-	-	503 (94.02)	580 (93.40)	-	-	-	1569 (95.32)	-	-	-
*No*	-	-	-	-	-	-	32 (5.98)	41 (6.60)	-	-	-	77 (4.68)	-	-	-
**In the past year, how many days were there usually between your periods?**															
*Mean (SD)*	-	-	-	-	-	-	-	-	28.35 (7.12)	27.81 (4.97)	27.62 (5.62)	-	27.32 (5.28)	-	-
< 15 days *	-	-	-	-	-	-	-	-	13 (1.39)	12 (1.31)	11 (0.91)	-	11 (0.89)	-	-
15–20 days *	-	-	-	-	-	-	-	-	18 (1.93)	12 (1.31)	37 (3.08)	-	14 (1.13)	-	-
21–25 days *	-	-	-	-	-	-	-	-	138 (14.79)	168 (18.36)	229 (19.04)	-	311 (25.20)	-	-
26–30 days *	-	-	-	-	-	-	-	-	636 (68.17)	610 (66.67)	791 (65.75)	-	773 (62.64)	-	-
31–35 days *	-	-	-	-	-	-	-	-	92 (9.86)	89 (9.73)	109 (9.06)	-	98 (7.94)	-	-
36–40 days *	-	-	-	-	-	-	-	-	12 (1.29)	12 (1.31)	14 (1.16)	-	16 (1.30)	-	-
> 40 days *	-	-	-	-	-	-	-	-	24 (2.57)	12 (1.31)	12 (1.00)	-	11 (0.89)	-	-
**In the last 3 months, have you had a period or menstrual bleeding?**															
*Yes*	-	-	-	-	-	-	-	-	-	-	-	-	-	1784 (82.75)	-
*No*	-	-	-	-	-	-	-	-	-	-	-	-	-	372 (17.25)	-
**If no, were your periods stopped by:**															
*Surgery*	-	-	-	-	-	-	-	-	-	-	-	-	-	<5	-
*Chemotherapy or radiation therapy*	-	-	-	-	-	-	-	-	-	-	-	-	-	<5	-
*Pregnancy or breastfeeding*	-	-	-	-	-	-	-	-	-	-	-	-	-	41 (12.85)	-
*No obvious reason / menopause*	-	-	-	-	-	-	-	-	-	-	-	-	-	10 (3.13)	-
*Contraception*	-	-	-	-	-	-	-	-	-	-	-	-	-	260 (81.50)	-
*Periods not started yet*	-	-	-	-	-	-	-	-	-	-	-	-	-	6 (1.88)	-
**What is the length of your usual menstrual cycle (the interval from first day of period to first day of next period), when you are NOT using oral contraception, injections, or implant?**															
*Mean (SD)*	-	-	-	-	-	-	-	-	-	-	-	-	-	-	25.74 (9.47)
< 15 days *	-	-	-	-	-	-	-	-	-	-	-	-	-	-	122 (11.87)
15–20 days *	-	-	-	-	-	-	-	-	-	-	-	-	-	-	13 (1.26)
21–25 days *	-	-	-	-	-	-	-	-	-	-	-	-	-	-	154 (14.98)
26–30 days *	-	-	-	-	-	-	-	-	-	-	-	-	-	-	645 (62.74)
31–35 days *	-	-	-	-	-	-	-	-	-	-	-	-	-	-	69 (6.71)
36–40 days *	-	-	-	-	-	-	-	-	-	-	-	-	-	-	11 (1.07)
> 40 days *	-	-	-	-	-	-	-	-	-	-	-	-	-	-	14 (1.36)
Derived variables															
*Period in last 3 months*	<5	6 (NA)	39 (NA)	300 (NA)	809 (NA)	1077 (NA)	503 (94.02)	580 (93.40)	930 (NA)	915 (NA)	1201 (NA)	1569 (95.32)	1233 (NA)	1784 (82.75)	1024 (NA)
*No period in last 3 months*	<5	<5	<5	<5	<5	<5	32 (5.98)	41 (6.60)	<5	<5	<5	77 (4.68)	<5	372 (17.25)	<5

CBQ: child-based questionnaire completed by mothers. Cell counts below 5 have been replaced with <5, and this may include 0. *Continuous responses recoded.

***Cycle regularity***

At the first three research clinics (11.7, 12.8, and 13.8 years), participants reported whether their periods were regular or not (or they didn’t know). At two later clinics (17.8 and 24 years) and two child-completed questionnaires (19.6 and 21 years), respondents were asked about the regularity or length of their cycle and, alongside a series of different cycle length options, were able to report that their cycle was not regular or too irregular for them to estimate their cycle length. In addition, in the mean age 21 questionnaire, participants were asked whether their periods were very, moderately, mildly, or not at all irregular. Table 11 provides an overview of these variables and summarises the responses of those who had started their periods.

**Table 11.** Original and derived G1 cycle regularity variables (N (%)).

Question	11.7 years clinic	12.8 years clinic	13.8 years clinic	17.8 years clinic	19.6 years	21 years	24 years clinic
Original variables							
**(Teenagers’) periods are regular**							
*Yes*	274 (51.99)	817 (49.13)	1309 (61.03)	-	-	-	-
*No*	155 (29.41)	504 (30.31)	560 (26.11)	-	-	-	-
*Don’t know*	98 (18.60)	342 (20.57)	276 (12.87)	-	-	-	-
**Periods are regular**							
*Yes, occur every 28–30 days*	-	-	-	1843 (66.80)	-	-	-
*Yes, occur less than every 28 days*	-	-	-	220 (7.97)	-	-	-
*Yes, occur more than every 30 days*	-	-	-	132 (4.78)	-	-	-
*No*	-	-	-	564 (20.44)	-	-	-
**Are your periods regular?**							
*Yes, every 23 days or less*	-	-	-	-	423 (22.23)	-	-
*Yes, between 24 and 35 days*	-	-	-	-	973 (51.13)	-	-
*Yes, > every 35 days*	-	-	-	-	47 (2.47)	-	-
*No*	-	-	-	-	460 (24.17)	-	-
**Are/were your periods irregular?**							
*Very*	-	-	-	-	-	326 (15.62)	-
*Moderately*	-	-	-	-	-	300 (14.37)	-
*Mildly*	-	-	-	-	-	443 (21.23)	-
*Not at all*	-	-	-	-	-	1018 (48.78)	-
**How many days do you usually have between the start of one period and the start of the next period?**							
*Less than 21 days*	-	-	-	-	-	103 (5.03)	-
*21–25 days*	-	-	-	-	-	697 (34.07)	-
*26–31 days*	-	-	-	-	-	752 (36.75)	-
*32–39 days*	-	-	-	-	-	115 (5.62)	-
*40–50 days*	-	-	-	-	-	38 (1.86)	-
*More than 50 days*	-	-	-	-	-	40 (1.96)	-
*Too irregular to estimate*	-	-	-	-	-	301 (14.71)	-
**Approximate length of usual menstrual cycle**							
*Less than 25 days*	-	-	-	-	-	-	154 (12.10)
*25–34 days*	-	-	-	-	-	-	833 (65.44)
*35–60 days*	-	-	-	-	-	-	72 (5.66)
*More than 60 days*	-	-	-	-	-	-	15 (1.18)
*Too irregular to estimate*	-	-	-	-	-	-	199 (15.63)
Derived variables							
*Regular*	274 (63.87)	817 (61.85)	1309 (70.04)	2195 (79.56)	1443 (75.83)	1448 (68.37)	1074 (84.37)
*Irregular*	155 (36.13)	504 (38.15)	560 (29.96)	564 (20.44)	460 (24.17)	670 (31.63)	199 (15.63)

***Heavy or prolonged bleeding***

In the nine puberty questionnaires, participants (or their mothers up to 13.1 years) were asked whether they had experienced heavy or prolonged bleeding with their period. Following this, participants who reported heavy or prolonged bleeding were asked whether they had contacted a doctor for this. Participants were also asked to report the number of days bleeding they normally experienced. If participants were unsure about the exact number of days, they could instead select one of three options: 3 days or less, 4–6 days, or 7 days or more. In the age 21 questionnaire, participants were asked whether their periods were very, moderately, mildly, or not at all heavy. Table 12 provides a summary of these variables and the responses amongst G1 participants who had started their periods.

**Table 12.** Original and derived G1 heavy and/or prolonged bleeding variables (N (%)).

Question	8.1 years	9.6 years	10.6 years	11.6 years	13.1 years	14.6 years	15.5 years	16 years	17 years	21 years
Original variables										
**Has your daughter ever had any of the following symptoms associated with their period: heavy or prolonged bleeding?**										
*Yes*	<5	<5	9 (12.33)	99 (18.97)	421 (21.92)	-	-	-	-	-
*No*	<5	13 (NA)	64 (87.67)	423 (81.03)	1500 (78.08)	-	-	-	-	-
**Have you had any of the following symptoms associated with your period: heavy or prolonged bleeding?**										
*Yes*	-	-	-	-	-	908 (34.15)	850 (34.34)	1007 (36.53)	920 (35.91)	-
*No*	-	-	-	-	-	1751 (65.85)	1625 (65.66)	1750 (63.47)	1642 (64.09)	-
**If yes, did you contact a doctor for this?**										
*Yes*	<5	<5	<5	22 (22.45)	56 (13.37)	128 (14.17)	145 (17.26)	268 (26.61)	289 (31.58)	-
*No*	<5	<5	6 (NA)	76 (77.55)	363 (86.63)	775 (85.83)	695 (82.74)	739 (73.39)	626 (68.42)	-
**In the past year, how many days of bleeding has your daughter usually had during each period?**										
*Mean (SD)*	2 (NA)	2.50 (1.78)	4.48 (1.93)	5.22 (1.66)	5.41 (1.44)	-	-	-	-	-
< 3 days *	<5	9 (75.00)	12 (19.05)	17 (3.97)	21 (1.37)	-	-	-	-	-
*3 days*	<5	<5	6 (9.52)	42 (9.81)	80 (5.24)	-	-	-	-	-
*4 days*	<5	<5	11 (17.46)	60 (14.02)	252 (16.49)	-	-	-	-	-
*5 days*	<5	<5	16 (25.40)	139 (32.48)	510 (33.38)	-	-	-	-	-
*6 days*	<5	<5	9 (14.29)	71 (16.59)	332 (21.73)	-	-	-	-	-
*7 days*	<5	<5	7 (11.11)	87 (20.33)	273 (17.87)	-	-	-	-	-
*8 days*	<5	<5	<5	6 (1.40)	39 (2.55)	-	-	-	-	-
*9 days*	<5	<5	<5	<5	8 (0.52)	-	-	-	-	-
> 9 days *	<5	<5	<5	5 (1.17)	13 (0.85)	-	-	-	-	-
**In the past year, how many days of bleeding have you usually had during each period?**										
*Mean (SD)*	-	-	-	-	-	5.54 (2.15)	5.38 (1.33)	5.39 (1.32)	5.31 (1.94)	-
< 3 days *	-	-	-	-	-	6 (0.36)	11 (0.69)	15 (0.78)	21 (1.11)	-
*3 days*	-	-	-	-	-	72 (4.29)	74 (4.62)	90 (4.66)	107 (5.68)	-
*4 days*	-	-	-	-	-	264 (15.74)	277 (17.29)	318 (16.48)	359 (19.05)	-
*5 days*	-	-	-	-	-	602 (35.90)	579 (36.14)	715 (37.05)	700 (37.14)	-
*6 days*	-	-	-	-	-	365 (21.77)	344 (21.47)	384 (19.90)	339 (17.98)	-
*7 days*	-	-	-	-	-	300 (17.89)	262 (16.35)	347 (17.98)	296 (15.70)	-
*8 days*	-	-	-	-	-	44 (2.62)	39 (2.43)	37 (1.92)	39 (2.07)	-
*9 days*	-	-	-	-	-	7 (0.42)	7 (0.44)	11 (0.57)	8 (0.42)	-
> 9 days *	-	-	-	-	-	17 (1.01)	9 (0.56)	13 (0.67)	16 (0.85)	-
**If you don’t know, is it probably:**										
*7 days or more*	<5	<5	<5	15 (11.72)	46 (9.89)	141 (11.52)	122 (11.52)	118 (11.71)	104 (12.46)	-
*4–6 days*	<5	<5	6 (50.00)	86 (67.19)	352 (75.70)	1016 (83.01)	884 (83.47)	826 (81.94)	656 (78.56)	-
*3 days or less*	<5	<5	6 (50.00)	27 (21.09)	67 (14.41)	67 (5.47)	53 (5.00)	64 (6.35)	75 (8.98)	-
**How heavy were/are your periods?**										
*Very*	-	-	-	-	-	-	-	-	-	312 (14.89)
*Moderately*	-	-	-	-	-	-	-	-	-	1060 (50.57)
*Mildly*	-	-	-	-	-	-	-	-	-	581 (27.72)
*Not at all*	-	-	-	-	-	-	-	-	-	143 (6.82)
Derived variables										
*Heavy or prolonged bleeding* **	<5	<5	10 (13.51)	100 (18.87)	429 (22.14)	919 (34.04)	852 (34.18)	1012 (36.44)	923 (35.90)	1372 (65.46)
*Neither heavy nor prolonged bleeding* **	<5	13 (NA)	64 (86.49)	430 (81.13)	1509 (77.86)	1781 (65.96)	1641 (65.82)	1765 (63.56)	1648 (64.10)	724 (34.54)

Cell counts below 5 have been replaced with <5, and this may include 0. *Continuous responses recoded. **Heavy bleeding is defined as reporting ‘heavy or prolonged bleeding’, more than 8 days bleeding, or ‘very’/‘moderately’ heavy periods, whereas not heavy is defined as reporting ‘no heavy or prolonged bleeding’, bleeding for 8 days or less, or periods that are ‘mildly’/’not at all’ heavy.

***Menstrual pain***

In the puberty questionnaires, except for 15.5 years, participants (or their mothers up to 13.1 years) were asked whether or not they had experienced severe cramps with their periods. At age 15.5, they were instead asked whether they had experienced pain with their periods, and then, for those who answered yes, whether the pain was severe, moderate, or mild. All puberty questionnaires also asked participants who reported severe cramps or pain with their periods whether they had contacted a doctor for this. In the age 21 questionnaire, participants were asked whether their periods were very, moderately, mildly, or not at all painful. Table 13 provides a summary of these variables.

**Table 13.** Original and derived G1 menstrual pain variables (N (%)).

Question	8.1 years	9.6 years	10.6 years	11.6 years	13.1 years	14.6 years	15.5 years	16 years	17 years	21 years
Original variables										
**Has your daughter ever had any of the following symptoms associated with her period: severe cramps?**										
*Yes*	<5	5 (33.33)	10 (15.87)	126 (26.30)	563 (32.13)	-	-	-	-	-
*No*	<5	10 (66.67)	53 (84.13)	353 (73.70)	1189 (67.87)	-	-	-	-	-
**Have you ever had any of the following symptoms associated with your period: severe cramps?**										
*Yes*	-	-	-	-	-	1271 (48.85)	-	1515 (56.32)	1451 (57.92)	-
*No*	-	-	-	-	-	1331 (51.15)	-	1175 (43.68)	1054 (42.08)	-
**Have you ever had any of the following symptoms associated with your period: pain with your period?**										
*Yes*	-	-	-	-	-	-	2108 (85.10)	-	-	-
*No*	-	-	-	-	-	-	369 (14.90)	-	-	-
**If so, were they mild, moderate, or severe?**										
*Severe*	-	-	-	-	-	-	405 (19.33)	-	-	-
*Moderate*	-	-	-	-	-	-	1114 (53.17)	-	-	-
*Mild*	-	-	-	-	-	-	576 (27.49)	-	-	-
**If yes, did you contact a doctor for this?**										
*Yes*	<5	<5	<5	14 (11.20)	49 (8.81)	148 (11.75)	194 (9.29)	327 (21.61)	371 (25.76)	-
*No*	<5	<5	6 (NA)	111 (88.80)	507 (91.19)	1112 (88.25)	1894 (90.71)	1186 (78.39)	1069 (74.24)	-
**How painful are/were your periods?**										
*Very*	-	-	-	-	-	-	-	-	-	327 (15.60)
*Moderately*	-	-	-	-	-	-	-	-	-	686 (32.73)
*Mildly*	-	-	-	-	-	-	-	-	-	785 (37.45)
*Not at all*	-	-	-	-	-	-	-	-	-	298 (14.22)
Derived variables										
Painful *	<5	<5	10 (15.87)	126 (26.30)	563 (32.13)	1271 (48.85)	1519 (61.65)	1515 (56.32)	1451 (57.92)	1013 (48.33)
Not painful *	<5	10 (NA)	53 (84.13)	353 (73.70)	1189 (67.87)	1331 (51.15)	945 (38.35)	1175 (43.68)	1054 (42.08)	1083 (51.67)

Cell counts below 5 have been replaced with <5, and this may include 0. *Painful periods are defined as reporting ‘severe cramps’, ‘severe/moderate pain with your period’, or ‘very/moderately’ painful periods, whereas not painful is defined as reporting ‘no severe cramps’, ‘mild/no pain with your period’, or periods that are ‘mildly/not at all’ painful.

***Premenstrual symptoms***

At 21 years only ( Table 14), participants were asked whether they experienced ‘particular problems’ in the days before or during their periods. Those who responded yes were subsequently asked which problems they experienced and were provided with multiple symptoms: very fatigued, irritable, depressed, anxious, or other. Respondents indicated whether they experienced each of these symptoms before their periods, during their periods, or not at all (participants were able to select more than one option).

**Table 14.** Original and derived G1 premenstrual symptoms variables (N (%)).

Question	21 years
Original variables	
**Do/did you generally find that in the days before or during your** ** periods you have particular problems?**	
*Yes*	1101 (52.23)
*No*	1007 (47.77)
**If yes, which problems do you experience: very fatigued?**	
*Yes, before*	438 (20.48)
*Yes, during*	460 (21.50)
*No, I don’t experience this*	425 (19.87)
**If yes, which problems do you experience: irritable?**	
*Yes, before*	790 (36.90)
*Yes, during*	523 (24.44)
*No, I don’t experience this*	124 (5.80)
**If yes, which problems do you experience: depressed?**	
*Yes, before*	466 (21.80)
*Yes, during*	324 (15.15)
*No, I don’t experience this*	482 (22.53)
**If yes, which problems do you experience: anxious?**	
*Yes, before*	274 (12.82)
*Yes, during*	188 (8.79)
*No, I don’t experience this*	723 (33.80)
**If yes, which problems do you experience: other?**	
*Yes, before*	288 (13.47)
*Yes, during*	154 (7.20)
*No, I don’t experience this*	374 (17.48)
Derived variables	
Binary variable: very fatigued *	
*Very fatigued*	668 (32.19)
*Not very fatigued*	1407 (67.81)
Binary variable: irritable *	
*Irritable*	986 (47.04)
*Not irritable*	1110 (52.96)
Binary variable: depressed *	
*Depressed*	613 (29.56)
*Not depressed*	1461 (70.44)
Binary variable: anxious *	
*Anxious*	365 (17.66)
*Not anxious*	1702 (82.34)
Binary variable: other symptoms *	
*Other symptoms*	288 (17.45)
*No other symptoms*	1362 (82.55)
**Number of PMS-related symptoms**	
*0*	998 (61.38)
*1*	138 (8.49)
*2*	189 (11.62)
*3*	161 (9.90)
*4*	92 (5.66)
*5*	48 (2.95)

*Participants are classified as experiencing the PMS-related symptom (very fatigued, irritable, depressed, anxious, or other) if they reported the symptom either before or during their period, whereas they are classified as not experiencing the symptom if they did not report the symptom before and during their period.

### Using the menstrual cycle feature data

Whilst few of the menstrual cycle feature variables are consistent across all available timepoints, it is possible to derive comparable variables for most features and timepoints. However, there are some important limitations that need to be considered when deriving such variables and planning analyses.

***Cycle length***

**Figure 3.**
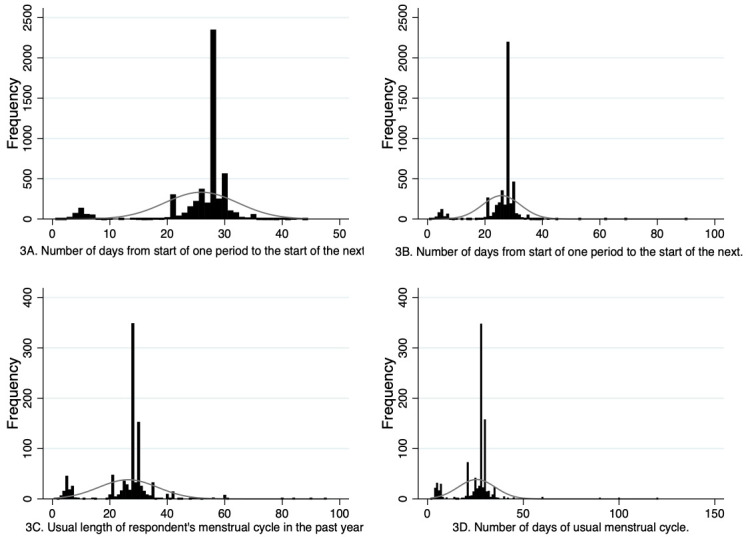
Histograms showing G0 and G1 cycle length variables. **3A** is from the G0 37.8 year questionnaire and **3B** is from the G0 40.3 year questionnaire. **3C** is from the G1 13.1 year puberty questionnaire and **3D** is from the G1 age 24 clinic assessment.

For both G0 and G1, participants reported the exact number of days of their menstrual cycle at multiple timepoints and this data could be used continuously or could be used to create categorical variables. For the continuous data, it is important to be aware of outliers in the data, with a small number of G0 participants reporting cycle lengths as long as 90 days and G1 participants reporting cycle lengths up to 150 days. It is challenging to know whether these responses reflect genuine long cycle lengths, for example reflecting an episode of amenorrhea, or if they are due to data entry errors or participants misunderstanding these questions. Approaches to managing these potential erroneous outliers will depend on the research questions being addressed and could include keeping all participants in the analyses and then repeating analyses with those whose cycle lengths are notably different to most participants (e.g. 4 standard deviations away from the mean), or some other threshold to see if the outlying values influence results, and reclassifying some participants at each reporting timepoint as having amenorrhea if they report a cycle length of 84 days or more (cessation of menstruation for three months). A further issue, however, is that of small peaks in the distribution around 5 days at some of the timepoints ( Figure 3), suggesting that some individuals are reporting the number of days bleeding rather than cycle length. This needs to be managed when deriving variables for analyses. One possible method is to use multiple imputation to impute responses less than a certain threshold (e.g., 10), with responses to the same question at other timepoints contributing to the imputation model. Similar to managing outliers, there will be multiple methods that could be adopted to handle these possible misinterpretations, and a sensitivity analysis to compare results with different approaches is advised. Moreover, the wording of the questions varied at different timepoints for the G1 sample, which may have impacted how participants responded. For example, some questions specified that the length of a usual menstrual cycle referred to the interval from the first day of period to the first day of next period whereas others did not provide this clarification. It is possible therefore that some participants may have reported the number of days between the end of the first period and the start of the next period at some timepoints, possibly impacting the accuracy of reporting and how appropriate it is to compare responses between timepoints. Researchers should consider how to manage this limitation when designing their analyses.

Researchers may want to derive categorical variables to describe infrequent, frequent, and normal cycles ( Table 2 and Table 9). Normal cycles are considered to range from 24–38 days, whereas cycles shorter than 24 days are considered frequent and those longer than 38 days are considered infrequent ^10^. Such variables could be derived from the continuous variables (noting the issues above). Unfortunately, although ALSPAC also collected categorical data at some timepoints, the original variable categories do not always align with the clinical definitions of infrequent/frequent/normal. Moreover, the boundaries vary between the data collection timepoints. At some G0 and G1 timepoints, the categories are 23 days or less, 24–35 days, and more than every 35 days, whereas at others, the categories are less than 21 days, 21–25 days, 26–31 days, 32–39 days, 40–50 days, and more than 50 days. Finally, at the age 24 timepoint for G1, the categories are less than 25 days, 25–34 days, 35–60 days, and more than 60 days. Therefore, it may not be plausible to include all the available timepoints in the desired analyses, depending on its nature and the timepoints of interest.

***Amenorrhea***

Binary variables can be derived for amenorrhea for both G0 and G1 (no period in at least the last 3 months vs. had a period in the last 3 months) at each timepoint ( Table 3 and Table 10). However, most respondents who reported not having a period in the last 3 months provided a reason (e.g., surgery, chemotherapy, pregnancy, menopause, contraception, or not started their periods yet). It is also possible that this may be the case at timepoints where participants were not asked to provide a reason. Information on contraception use and other possible explanations is available from other data collection waves but not necessarily from same wave in which information on amenorrhea was collected (see further considerations below). This could be utilised to attempt to infer whether a participant who has reported not having a period in the last 3 months was also on contraception that could have explained this.

The cycle length variables whereby participants reported the exact number of days of their cycles could also be utilised. To be consistent with the above variables, the cycle length variables could be used to derive binary variables reflecting cycles of 84 days or more (i.e., no period in the last 3 months) compared with cycles of less than 84 days. This would be consistent with clinical guidelines which characterise amenorrhea, specifically secondary amenorrhea, as the cessation of menstruation for three months ^13, 22^. However, as discussed above, it is possible that the high values reported by participants in response to questions about their cycle length could be the result of data entry errors and therefore researchers should be cautious including these variables.

***Cycle regularity***

For both G0 and G1, the cycle regularity data could be used to derive a binary variable at each timepoint (regular vs. irregular) whereby a participant is classified as having irregular periods if they reported that their period was not regular, too irregular to estimate their cycle length, or very/moderately irregular ( Table 4 and Table 11). Alternatively, the more granular 4-level variables for G0 (very, moderately, mildly, or not at all irregular) could be maintained as they are available at half of the timepoints (mean age 32.0 33.1, 34.0, 35.1, 38.3, 41.4, 48.6, and 51.3 years) if these are the only timepoints being considered in the analysis.

***Heavy or prolonged bleeding***

For the G0 sample, granular 4-level heavy bleeding variables (very, moderately, mildly, or not at all) could be utilised as these are available at each timepoint or binary variables could be derived (very or moderately heavy vs. mildly or not at all heavy). Also, at each of these timepoints, participants reported the duration of bleeding. Binary variables for prolonged bleeding, which is defined as more than 8 days bleeding ^10^, could therefore also be derived (>8 days bleeding vs. 8 days bleeding or less) ( Table 5).

For G1 however, it is not possible to separate heavy and prolonged bleeding in line with clinical guidelines due to the questions that were asked ^10^. Instead, binary variables for heavy or prolonged bleeding could be derived (heavy or prolonged bleeding vs. neither heavy nor prolonged bleeding), whereby those who reported heavy or prolonged bleeding, very or moderately heavy bleeding, or bleeding for more than 8 days are classified as having heavy or prolonged bleeding ( Table 12). Unfortunately, the G1 categorical response variables for days bleeding (3 days or less, 4–6 days, or 7 days or more) do not reflect the clinical guidelines regarding prolonged bleeding so are more challenging to incorporate into this binary definition. It would also be possible to derive the same binary variables for the G0 sample if it was more appropriate for the specific research question to have comparable variables across the two generations ( Table 5).

A key consideration with regards to the number of days bleeding variables is that there are some outliers. For example, at the G0 mean age 41.4 year questionnaire and the aged 14.6 and 17 G1 puberty questionnaire, participants reported up to 60 days bleeding. These outliers could be due to data entry errors, participants misunderstanding the question as relating to intervals between periods, or they could be genuine responses reflecting very long periods of bleeding. It is not possible with the available data to distinguish the reasons for such high values and therefore there is no clear way to handle such outliers. Approaches could include recoding such values (e.g., 4 SDs from the mean or the 99th percentile) as missing, imputing them based on available data at other timepoints, or replacing them with the highest non-outlier value (‘top-coding’). The most appropriate method will depend on the nature of the analyses being conducted; however, it may be beneficial to conduct a sensitivity analysis to compare the results with different approaches to handling the outliers.

***Menstrual pain***

It is possible to derive binary pain variables for both G0 and G1 ( Table 6 and Table 13). Most of the G1 pain-related variables are already in a binary format except for the age 15 and 21 ones. These variables could be dichotomised whereby those reporting severe or moderate pain are categorised as having pain associated with their periods. The G0 variables could be dichotomised whereby those reporting very/moderately painful periods could be categorised as having pain associated with their periods, or these could be utilised as 4-level variables if appropriate for the analysis to maintain granularity.

However, some caution is needed regarding the G1 variables as the differences in the wording of the questions may mean that participants responded differently to the binary puberty questions. The majority of the puberty questions ask about ‘severe cramps’ whereas the 15.5 puberty question asks about ‘pain with your period’ and, subsequently, whether these are severe, moderate, or mild. These are slightly different concepts, and this does appear to be reflected in the responses as the preceding and following timepoint have 48.9% and 56.3% of people reporting severe cramps respectively, but only 16.4% of all respondents at 15.5 report severe pain and 61.7% report severe or moderate pain.

***Premenstrual symptoms***

There are multiple ways in which the PMS-related variables could be used depending on the nature of analysis. Firstly, a binary variable could be derived which reflects experiencing ‘particular problems’, ‘problems with their period’, ‘pre-menstrual tension’, or any of the listed problems related to periods (irritable, anxious, depressed, very fatigued, other) vs no problems. Secondly, binary variables could be derived for each of these symptoms separately (e.g., irritable vs not irritable). These variables could be derived separately or together for before and during the period. This would be useful for examining the timing of specific symptoms and for distinguishing symptoms occurring during a period from those occurring before, in line with clinical definitions of PMS ^23, 24^. Finally, it would also be possible to derive a variable reflecting the number of PMS-related symptoms an individual experiences, either before their periods only (in line with clinical definitions of PMS) or during a period ( Table 7 and Table 14). Whilst PMS is not defined by a specific number of symptoms, this variable, which would range from 0 (no PMS symptoms) to 5 (all listed PMS symptoms), could be useful to examine the severity of PMS as more symptoms may reflect a greater negative impact on daily functioning ^23, 24^.

A key consideration for the PMS-related data is that PMS encompasses a wide range of physical and psychological symptoms. There are additional common PMS-related symptoms, such as headaches, acne, and breast tenderness ^23, 24^, which are not reported in the ALSPAC data. Whilst there is an option for participants to report other problems with their period, we cannot be sure how participants have interpreted this, nor that people have reported all other relevant symptoms. A further issue with the G0 data is that some of the earlier timepoints only ask about ‘particular problems’ but are not followed up with questions about which symptoms are experienced and their timing. A broad range of problems could be labelled by participants as ‘particular problems’, not all of which would necessarily be considered PMS. Therefore, there may be some misclassification. Misclassification may also arise due to the retrospective nature of such assessments, which have been suggested to result in an overreporting of symptoms compared with prospective diary reporting ^25^. Finally, the G1 data is somewhat limited because it is only available at one timepoint.

### Further considerations

Contraception is a primary factor that must be considered when utilising the menstrual cycle feature data outlined above. Hormonal contraception is particularly prevalent and, although participants on hormonal contraception will not experience natural menstrual cycles, some will experience withdrawal bleeds that they may classify as menstrual periods (and respond to questions about their menstrual cycle features accordingly). In addition, hormonal contraception is often prescribed in response to menstrual cycle issues such as heavy menstrual bleeding, irregular cycles, or pain. Therefore, as menstrual cycle features and contraception are bidirectionally associated, it may be inappropriate to adjust for or exclude those on hormonal contraception depending on the analyses being conducted. For example, research examining whether BMI influences heavy menstrual bleeding would not want to exclude individuals on hormonal contraception as BMI can impact the likelihood of using hormonal contraception. Excluding would therefore result in collider bias and potentially result in a spurious association between BMI and heavy menstrual bleeding. There are other methods that may account for hormonal contraception when conducting analyses such as this, possibly including multiple imputation, meta-regression, and probability weighting, and there is data available on contraception in ALSPAC to enable this. Table 15 and Table 16 provide a summary of the available contraceptive variables for G0 and G1 respectively.

**Table 15.** G0 contraception variables (N (%)).

Question	29.2 years	30.0 years	32.0 years	33.1 years	34.0 years	35.1 years	37.8 years	38.3 years	39.3 years	40.3 years	41.4 years	47.4 years clinic	48.6 years	49.7 years	50.3 years clinic	51.3 years	51.6 years clinic	52.6 years clinic	57.7 years
**Since the baby was born / toddler was 8 months old / child was 18 months old / in the past year / child’s 5 ^th^ birthday / in the last 2 years, how often have you used the following: contraceptive pill?**																			
*Every day*	4625 (40.99)	3414 (33.03	2655 (27.55)	2447 (25.60)	2066 (2.98)	1765 (20.63)	-	1229 (15.47)	-	-	-	-	262 (6.63)	-	-	-	-	-	-
*Often*	450 (3.99)	611 (5.91)	727 (7.54)	416 (4.35)	296 (3.29)	271 (3.17)	-	190 (2.39)	-	-	-	-	32 (0.81)	-	-	-	-	-	-
*Sometimes*	677 (6.00)	726 (7.02)	795 (8.25)	478 (5.00)	344 (3.83)	276 (3.23)	-	213 (2.68)	-	-	-	-	36 (0.91)	-	-	-	-	-	-
*Not at all*	5530 (49.02)	5585 (54.03)	5460 (56.66)	6216 (65.04)	6286 (69.91)	6243 (72.97)	-	6313 (79.46)	-	-	-	-	3624 (91.65)	-	-	-	-	-	-
**What forms of contraception are you using now?**																			
*Withdrawal*	-	204 (2.35)	248 (2.60)	237 (2.80)	-	-	-	-	-	-	-	-	-	-	-	-	-	-	-
*The pill*	-	3409 (39.31)	2964 (31.13)	2635 (31.08)	-	-	-	-	-	-	-	-	-	-	-	-	-	-	-
*IUCD / coil*	-	750 (8.65)	757 (7.95)	831 (9.80)	-	-	-	-	-	-	-	-	-	-	-	-	-	-	-
*Condom / sheath*	-	1861 (21.47)	1767 (18.56)	1863 (21.97)	-	-	-	-	-	-	-	-	-	-	-	-	-	-	-
*Calendar / rhythm method*	-	138 (1.59)	137 (1.44)	183 (2.16)	-	-	-	-	-	-	-	-	-	-	-	-	-	-	-
*Diaphragm / cap*	-	149 (1.72)	108 (1.13)	101 (1.19)	-	-	-	-	-	-	-	-	-	-	-	-	-	-	-
*Spermicide*	-	158 (1.82)	156 (1.64)	131 (1.55)	-	-	-	-	-	-	-	-	-	-	-	-	-	-	-
*None*	-	1272 (14.67)	2272 (23.86)	885 (10.44)	-	-	-	-	-	-	-	-	-	-	-	-	-	-	-
*Other*	-	728 (8.40)	1113 (11.69)	1612 (19.01)	-	-	-	-	-	-	-	-	-	-	-	-	-	-	-
**What forms of contraception are you using now?**																			
*Withdrawal*	-	-	-	-	225 (2.86)	231 (2.96)	-	207 (2.78)	-	177 (2.50)	190 (2.90)	-	84 (2.57)	-	-	-	-	-	-
*The pill*	-	-	-	-	2221 (26.49)	1953 (25.01)	-	1247 (16.72)	-	932 (13.14)	809 (12.36)	-	252 (7.70)	-	-	-	-	-	-
*IUCD / coil*	-	-	-	-	858 (10.23)	856 (10.96)	-	848 (11.37)	-	873 (12.31)	824 (12.59)	-	551 (16.83)	-	-	-	-	-	-
*Condom / sheath*	-	-	-	-	1640 (19.56)	1480 (18.95)	-	1126 (15.10)	-	968 (13.65)	821 (12.55)	-	330 (10.08)	-	-	-	-	-	-
*Calendar / rhythm method*	-	-	-	-	178 (2.12)	242 (3.10)	-	101 (1.35)	-	91 (1.28)	78 (1.19)	-	40 (1.22)	-	-	-	-	-	-
*Diaphragm / cap*	-	-	-	-	85 (1.01)	41 (0.53)	-	43 (0.58)	-	32 (0.45)	36 (0.55)	-	8 (0.24)	-	-	-	-	-	-
*Spermicide*	-	-	-	-	100 (1.19)	137 (1.75)	-	54 (0.72)	-	34 (0.48)	37 (0.57)	-	15 (0.46)	-	-	-	-	-	-
*I have been sterilised / am no longer fertile*	-	-	-	-	676 (8.06)	737 (9.44)	-	983 (13.18)	-	985 (13.89)	1025 (15.67)	-	421 (12.86)	-	-	-	-	-	-
*My partner has been sterilised*	-	-	-	-	1221 (14.57)	1383 (17.71)	-	1942 (26.05)	-	2060 (29.05)	1949 (29.79)	-	1047 (31.99)	-	-	-	-	-	-
*None*	-	-	-	-	816 (9.73)	434 (5.56)	-	660 (8.85)	-	613 (8.64)	575 (8.79)	-	399 (12.19)	-	-	-	-	-	-
*Other*	-	-	-	-	363 (4.33)	315 (4.03)	-	245 (3.29)	-	327 (4.61)	199 (3.04)	-	126 (3.85)	-	-	-	-	-	-
**Have you ever used the contraceptive pill?**																			
*Yes*	-	-	-	-	-	-	7410 (94.85)	-	-	7169 (94.12)	-	-	-	-	-	-	-	-	-
*No*	-	-	-	-	-	-	402 (5.15)	-	-	448 (5.88)	-	-	-	-	-	-	-	-	-
**Are you on the pill now?**																			
*Yes*	-	-	-	-	-	-	1402 (18.59)	-	-	999 (13.91)	-	-	-	-	-	-	-	-	-
*No*	-	-	-	-	-	-	6138 (81.41)	-	-	6184 (86.09)	-	-	-	-	-	-	-	-	-
**Have you used any medicines in the last 12 months for: oral contraceptive?**																			
*Yes*	-	-	-	-	-	-	-	-	1184 (14.54)	-	788 (11.08)	-	-	-	-	-	-	-	-
*No*	-	-	-	-	-	-	-	-	6969 (85.46)	-	6321 (88.92)	-	-	-	-	-	-	-	-
**Are you currently taking oral contraceptives?**																			
*Yes*	-	-	-	-	-	-	-	-	-	-	-	336 (6.69)	-	-	149 (4.98)	120 (3.83)	-	-	101 (3.33)
*No*	-	-	-	-	-	-	-	-	-	-	-	4686 (93.31)	-	-	2841 (95.02)	3011 (96.17)	-	-	2933 (96.67)
**Are you currently using contraceptive injection?**																			
*Yes*	-	-	-	-	-	-	-	-	-	-	-	42 (0.84)	-	-	39 (1.31)	31 (0.99)	-	-	25 (0.82)
*No*	-	-	-	-	-	-	-	-	-	-	-	4980 (99.16)	-	-	2936 (98.69)	3100 (99.01)	-	-	3009 (99.18)
**Are you currently using:**																			
*Oral contraceptive pill*	-	-	-	-	-	-	-	-	-	-	-	-	-	268 (27.10)	-	-	-	-	-
*Contraceptive injection*	-	-	-	-	-	-	-	-	-	-	-	-	-	36 (3.64)	-	-	-	-	-
*Contraceptive implant*	-	-	-	-	-	-	-	-	-	-	-	-	-	27 (2.73)	-	-	-	-	-
*Contraceptive coil with hormone*	-	-	-	-	-	-	-	-	-	-	-	-	-	653 (66.03)	-	-	-	-	-
*Contraceptive patch*	-	-	-	-	-	-	-	-	-	-	-	-	-	5 (0.51)	-	-	-	-	-
**What forms of contraception are you and your partner using now? (please cross all that you have used in the past 3 months)**																			
Withdrawal	-	-	-	-	-	-	-	-	-	-	-	-	-	-	-	78 (1.81)	-	-	38 (0.80)
The pill	-	-	-	-	-	-	-	-	-	-	-	-	-	-	-	201 (4.68)	-	-	56 (1.19)
IUD (coil, no hormones)	-	-	-	-	-	-	-	-	-	-	-	-	-	-	-	111 (2.58)	-	-	41 (0.87)
IUD (coil, with hormones, such as a mirena coil)	-	-	-	-	-	-	-	-	-	-	-	-	-	-	-	527 (12.26)	-	-	310 (6.56)
Condom / sheath	-	-	-	-	-	-	-	-	-	-	-	-	-	-	-	250 (5.82)	-	-	89 (1.88)
Calendar / rhythm method																21 (0.49)			<5
Diaphragm / cap	-	-	-	-	-	-	-	-	-	-	-	-	-	-	-	<5	-	-	<5
Spermicide	-	-	-	-	-	-	-	-	-	-	-	-	-	-	-	11 (0.26)	-	-	<5
Contraceptive injection	-	-	-	-	-	-	-	-	-	-	-	-	-	-	-	29 (0.67)	-	-	11 (0.23)
Contraceptive implant	-	-	-	-	-	-	-	-	-	-	-	-	-	-	-	31 (0.72)	-	-	11 (0.23)
I have been sterilised	-	-	-	-	-	-	-	-	-	-	-	-	-	-	-	335 (7.79)	-	-	189 (4.00)
My partner has been sterilised	-	-	-	-	-	-	-	-	-	-	-	-	-	-	-	781 (18.17)	-	-	277 (5.86)
I am no longer fertile	-	-	-	-	-	-	-	-	-	-	-	-	-	-	-	853 (19.84)	-	-	2156 (45.65)
None	-	-	-	-	-	-	-	-	-	-	-	-	-	-	-	734 (17.07)	-	-	1371 (29.03)
Other	-	-	-	-	-	-	-	-	-	-	-	-	-	-	-	333 (7.75)	-	-	174 (3.68)

**Table 16.** G1 contraception variables (N (%)).

Question	8.1 years	9.6 years	10.6 years	11.6 years	13.1 years	14.6 years	15.5 years	16 years	17 years	17.8 years clinic	19.6 years	21 years	24 years clinic
**Has your daughter taken oral contraceptives or birth control pills for any reason during the past 12 months?**													
*Yes*	<5	<5	<5	5 (0.96)	22 (1.13)	-	-	-	-	-	-	-	-
*No*	<5	15 (NA)	75 (NA)	518 (99.04)	1927 (98.87)	-	-	-	-	-	-	-	-
**Have you taken oral contraceptives or birth control pills for any reason during the past 12 months?**													
*Yes*	-	-	-	-	-	176 (6.55)	327 (13.16)	695 (25.20)	1110 (43.38)	-	-	-	-
*No*	-	-	-	-	-	2490 (92.70)	2139 (86.11)	2044 (74.11)	1435 (56.08)	-	-	-	-
*Don’t know*	-	-	-	-	-	18 (0.72)	18 (0.72)	19 (0.69)	14 (0.55)	-	-	-	-
**Currently taking contraception / Are you currently using**													
*Oral contraceptive* *pill*	-	-	-	-	-	-	-	-	-	1069 (73.83)	1015 (52.97)	-	-
*Contraceptive injection*	-	-	-	-	-	-	-	-	-	115 (7.94)	74 (3.86)	-	-
*Contraceptive implant*	-	-	-	-	-	-	-	-	-	33 (2.28)	136 (7.10)	-	-
*Contraceptive coil with hormone*	-	-	-	-	-	-	-	-	-	7 (0.48)	25 (1.30)	-	-
*Contraceptive patch*	-	-	-	-	-	-	-	-	-	<5	5 (0.26)	-	-
*None*	-	-	-	-	-	-	-	-	-	221 (15.26)	661 (34.50	-	-
**Which method of contraception (if any) are you or your sexual partner currently using? Cross true or false for each option**													
*I do not currently have a sexual partner*	-	-	-	-	-	-	-	-	-	-	-	392 (11.88)	-
*Not using any contraception*	-	-	-	-	-	-	-	-	-	-	-	443 (13.42)	-
*I / My partner has been sterilised*	-	-	-	-	-	-	-	-	-	-	-	<5	-
*Mini pill*	-	-	-	-	-	-	-	-	-	-	-	150 (4.55)	-
*Combined pill*	-	-	-	-	-	-	-	-	-	-	-	519 (15.73)	-
*Pill – not sure which*	-	-	-	-	-	-	-	-	-	-	-	759 (23.00)	-
*Mirena coil*	-	-	-	-	-	-	-	-	-	-	-	72 (2.18)	-
*Coil / other device*	-	-	-	-	-	-	-	-	-	-	-	55 (1.67)	-
*Condom*	-	-	-	-	-	-	-	-	-	-	-	354 (10.73)	-
*Femidom*	-	-	-	-	-	-	-	-	-	-	-	<5	-
*Cap / diaphragm*	-	-	-	-	-	-	-	-	-	-	-	<5	-
*Foams / gels / sprays / pessaries*	-	-	-	-	-	-	-	-	-	-	-	<5	-
*Contraceptive sponge*	-	-	-	-	-	-	-	-	-	-	-	<5	-
*Persona*	-	-	-	-	-	-	-	-	-	-	-	<5	-
*Safe period / rhythm method*	-	-	-	-	-	-	-	-	-	-	-	5 (0.15)	-
*Withdrawal*	-	-	-	-	-	-	-	-	-	-	-	80 (2.42)	-
*Injection*	-	-	-	-	-	-	-	-	-	-	-	91 (2.76)	-
*Implant*	-	-	-	-	-	-	-	-	-	-	-	273 (8.27)	-
*Emergency contraception*	-	-	-	-	-	-	-	-	-	-	-	49 (1.48)	-
*Going without sex*	-	-	-	-	-	-	-	-	-	-	-	42 (1.27)	-
*Don’t know / not sure*	-	-	-	-	-	-	-	-	-	-	-	<5	-
*Another method of contraception*	-	-	-	-	-	-	-	-	-	-	-	5 (0.15)	-
**Method of contraception used**													
*Combined pill*	-	-	-	-	-	-	-	-	-	-	-	-	867 (36.72)
*Progesterone only pill*	-	-	-	-	-	-	-	-	-	-	-	-	200 (8.47)
*Contraceptive injection*	-	-	-	-	-	-	-	-	-	-	-	-	91 (3.85)
*Contraceptive implant*	-	-	-	-	-	-	-	-	-	-	-	-	282 (11.94)
*Contraceptive patch*	-	-	-	-	-	-	-	-	-	-	-	-	8 (0.34)
*Coil*	-	-	-	-	-	-	-	-	-	-	-	-	95 (4.02)
*Hormonal coil / vaginal ring*	-	-	-	-	-	-	-	-	-	-	-	-	111 (4.70)
*Barrier methods*	-	-	-	-	-	-	-	-	-	-	-	-	149 (6.31)
*Natural family planning*	-	-	-	-	-	-	-	-	-	-	-	-	27 (1.14)
*None*	-	-	-	-	-	-	-	-	-	-	-	-	531 (22.49)

ALSPAC has collected data on multiple other reproductive factors that can influence menstrual cycle features, and researchers should consider these when planning analyses. Pregnancies and breastfeeding will result in absences of menstruation and can result in more problematic periods upon their initial resumption. Also, menopause and surgeries such as hysterectomies and oophorectomies will stop menstruation and therefore researchers will need to consider how to account for this. Age at menarche is another important factor assessed in ALSPAC which some researchers may want to consider depending on their research question. Whilst ALSPAC participants have been asked about recent pregnancies, breastfeeding, menopause, surgeries and hormonal contraceptive use, the questions are not necessarily asked at the same timepoint as the menstrual cycle features and therefore including such variables may require making inferences backwards or forwards in time.

Moreover, many of the menstrual cycle questions ask participants about their most recent period. Whilst most participants are likely to be answering such questions regarding a period they had up to one month ago, others may be answering about periods much further back in time prior to going on hormonal contraception, becoming pregnant, having a hysterectomy or oophorectomy or going through menopause. This further highlights the importance of considering other reproductive factors where possible to ensure only participants who are experiencing a menstrual cycle at the time of reporting are included in analyses. This is also important to consider as recall bias may become an issue for those who are answering questions about menstrual cycles that were experienced many months or years ago.

There is some limited data available on health conditions that might cause the problematic menstrual cycle features summarised in this data note. For example, participants were asked whether they had ever been diagnosed with PCOS at age 22 for G1 and mean age 49.7 for G0. The same question was asked about endometriosis at age 22 for G1 only. These data provide an opportunity to identify individuals whose problematic menstrual cycle features at previous timepoints are a result of one of these underlying disorders. However, as these conditions tend to be both underdiagnosed and take a long time to be diagnosed, the data is unlikely to capture all participants whose menstrual cycle features are due to either PCOS or endometriosis. There are many other factors which ALSPAC has detailed data on and, depending on the research question, researchers may wish to consider alongside menstrual features.

Missing data needs to be considered when using menstrual symptom data in ALSPAC. Missing data, for example due to loss to follow up, or participants not responding to some questions, could lead to selection bias if missingness is related to the exposure and outcome being explored ^26^. Missing menstrual symptom data could be due to a variety of reasons, including withdrawing from ALSPAC, loss to follow-up, not wanting to answer some questions, pregnancy, breastfeeding, menopause, contraception, and surgeries. It is plausible that several of these (e.g., withdrawal, loss to follow-up, pregnancy, breastfeeding) are socially patterned and hence likely to relate to the exposures and outcomes that are being related to the menstrual cycle features and therefore selection bias is possible. How this is explored and dealt with will depend on the specific research question being addressed and the pattern and extent of missing data. There are several papers that can help with exploring this, including ones that have been used previously in ALSPAC studies ^27, 28^.

One of the primary benefits of the ALSPAC dataset is the repeated measures of menstrual cycle features at multiple timepoints. This may allow researchers to increase their sample size by maintaining participants who have missing data at one timepoint by combining with data from other similar timepoints with multiple imputation. Beyond this, repeated measures can enable trajectory modelling to explore the causes and consequences of different patterns of menstrual cycle features over time if appropriate for the research question. The longitudinal nature of the data enables it to be utilised to assess causal relationships between menstrual cycle features and possible causes and consequences, reducing the likelihood of reverse causality.

However, researchers should also consider the possibility of misclassification. Whilst random measurement error is always a possibility, researchers should consider possible sources of systematic measurement error. For example, particular groups may feel more uncomfortable answering questions regarding their menstrual cycle and therefore provide answers that do not reflect their menstrual cycle features, or certain groups may be more or less likely to notice or report their menstrual cycle features. The subjective nature of many of the questions may also contribute to possible misclassification as participants may have different perspectives as to what constitutes certain symptoms, such as “heavy bleeding” or “severe cramps”. However, subjective experience of these features is crucial and is considered in the clinical guidelines for diagnosis of HMB ^10^. Moreover, menstrual cycle features may vary randomly over time, contributing to random error and the possibility of regression dilution when using repeated measures ^29^. Researchers therefore need to be aware of both random and systematic measurement error and address this as much as possible within their analysis.

## Data Availability

ALSPAC data access is through a system of managed open access. The steps below highlight how to apply for access to the data included in this data note and all other ALSPAC data. The datasets presented in this article are linked to ALSPAC project number B4175; please quote this project number during your application. The ALSPAC variable codes highlighted in the dataset descriptions can be used to specify required variables. 1. Please read the ALSPAC access policy which describes the process of accessing the data and samples in detail, and outlines the costs associated with doing so. 2. You may also find it useful to browse our fully searchable research proposal database which lists all research projects that have been approved since April 2011. 3. Please submit your research proposal for consideration by the ALSPAC Executive Committee. You will receive a response within 10 working days to advise you whether your proposal has been approved. If you have any questions about accessing data, please email alspac-data@bristol.ac.uk. The study website also contains details of all the data that is available through a fully searchable data dictionary.
