# Supplementary material for: Menstrual cycle features in mothers and daughters in the Avon Longitudinal Study of Parents and Children (ALSPAC)
Source: Wellcome Open Res. 2023 Nov 24;8:386. Originally published 2023 Sep 1. [Version 3] doi: 10.12688/wellcomeopenres.19774.3 (PMC10665607; doi:10.12688/wellcomeopenres.19774.3)
Supplement: Peer review history

# Reviewer report: Reviewer response for version 3

**DOI:** 10.21956/wellcomeopenres.22766.r70508

**Published:** 2023-11-27

**Authors:** Mandikudza Tembo

**Affiliations:** 1 Department of Global Health and Development, Faculty of Public Health and Policy, London School of Hygiene and Tropical Medicine, London, UK

Approved

Are sufficient details of methods and materials provided to allow replication by others?

Partly

Is the rationale for creating the dataset(s) clearly described?

Yes

Are the datasets clearly presented in a useable and accessible format?

Yes

Are the protocols appropriate and is the work technically sound?

Yes

Reviewer Expertise:

Menstrual health

I confirm that I have read this submission and believe that I have an appropriate level of expertise to confirm that it is of an acceptable scientific standard.

# Reviewer report: Reviewer response for version 2

**DOI:** 10.21956/wellcomeopenres.22386.r68897

**Published:** 2023-11-22

**Authors:** Jenny Doust

**Affiliations:** 1 The University of Queensland, Brisbane, Queensland, Australia

Thank you for responding to the comments that I made. This is a useful piece of work summarising the data available in the ALSPAC data set

Are sufficient details of methods and materials provided to allow replication by others?

Yes

Is the rationale for creating the dataset(s) clearly described?

Yes

Are the datasets clearly presented in a useable and accessible format?

Yes

Are the protocols appropriate and is the work technically sound?

Yes

Reviewer Expertise:

Women's health  Epidemiology

I confirm that I have read this submission and believe that I have an appropriate level of expertise to confirm that it is of an acceptable scientific standard.

# Reviewer report: Reviewer response for version 2

**DOI:** 10.21956/wellcomeopenres.22386.r68896

**Published:** 2023-10-25

**Authors:** Mandikudza Tembo

**Affiliations:** 1 Department of Global Health and Development, Faculty of Public Health and Policy, London School of Hygiene and Tropical Medicine, London, UK

Thank you for the changes made. I have no further comments to make.

Are sufficient details of methods and materials provided to allow replication by others?

Partly

Is the rationale for creating the dataset(s) clearly described?

Yes

Are the datasets clearly presented in a useable and accessible format?

Yes

Are the protocols appropriate and is the work technically sound?

Yes

Reviewer Expertise:

Menstrual health

I confirm that I have read this submission and believe that I have an appropriate level of expertise to confirm that it is of an acceptable scientific standard.

# Reviewer report: Reviewer response for version 1

**DOI:** 10.21956/wellcomeopenres.21901.r67570

**Published:** 2023-10-10

**Authors:** Jenny Doust

**Affiliations:** 1 The University of Queensland, Brisbane, Queensland, Australia

This paper outlines the data recorded on menstrual cycle features for mothers and index daughters in the Avon Longitudinal Study of Parents and Children. The multi-generational and longitudinal nature of the data makes it valuable to researchers.

 The data note helpfully outlines what data has been collected over what period of time and by what methods. It is particularly helpful to researchers in outlining some of the difficulties and pitfalls when using this data. These aspects of the data appear to have been carefully thought through in this report. The authors also provide information on how to apply to use this data for research.

 I have some minor comments for improving the paper. In the abstract, it would be helpful to understand the ages of the mothers and daughters. Currently it says that the data are collected at 21 and 20 timepoints but does not describe the ages of the participants. Secondly, in the description of the G1 cohort, for example in Figure 2, it is not clear if the ages described are the exact age of the participants or are the average ages.

Are sufficient details of methods and materials provided to allow replication by others?

Yes

Is the rationale for creating the dataset(s) clearly described?

Yes

Are the datasets clearly presented in a useable and accessible format?

Yes

Are the protocols appropriate and is the work technically sound?

Yes

Reviewer Expertise:

Women's health  Epidemiology

I confirm that I have read this submission and believe that I have an appropriate level of expertise to confirm that it is of an acceptable scientific standard, however I have significant reservations, as outlined above.

# Response

**Published:** 2023-11-21

**Authors:** Gemma Sawyer

**Affiliations:** University of Bristol, Bristol, England, UK

*We are grateful to the reviewer for their useful suggestions to improve the paper. Details of specific changes are outlined below (in italics), and we hope these address the reviewers’ critiques.*  

 This paper outlines the data recorded on menstrual cycle features for mothers and index daughters in the Avon Longitudinal Study of Parents and Children. The multi-generational and longitudinal nature of the data makes it valuable to researchers. The data note helpfully outlines what data has been collected over what period of time and by what methods. It is particularly helpful to researchers in outlining some of the difficulties and pitfalls when using this data. These aspects of the data appear to have been carefully thought through in this report. The authors also provide information on how to apply to use this data for research. I have some minor comments for improving the paper. In the abstract, it would be helpful to understand the ages of the mothers and daughters. Currently it says that the data are collected at 21 and 20 timepoints but does not describe the ages of the participants.

*We have added information on the ages of the mothers and daughters across the timepoints in the abstract. *  

 Secondly, in the description of the G1 cohort, for example in Figure 2, it is not clear if the ages described are the exact age of the participants or are the average ages.

*We have changed the X axis label in both Figure 1 and Figure 2 to clarify that these are the average (mean) ages of the G0 or G1 sample at each data collection timepoint.*  

 Is the rationale for creating the dataset(s) clearly described? Yes  

 Are the protocols appropriate and is the work technically sound? Yes  

 Are sufficient details of methods and materials provided to allow replication by others? Yes  

 Are the datasets clearly presented in a useable and accessible format? Yes

# Reviewer report: Reviewer response for version 1

**DOI:** 10.21956/wellcomeopenres.21901.r66481

**Published:** 2023-09-19

**Authors:** Mandikudza Tembo

**Affiliations:** 1 Department of Global Health and Development, Faculty of Public Health and Policy, London School of Hygiene and Tropical Medicine, London, UK

**Abstract**

 Overall, the abstract and key messages read very well. However, it would be helpful if the abstract noted where (geographical location) and when (time period including month and year) this research took place.

**Introduction**

 Very well written. However, please note the following edits:

- Citation missing for the first line.
- Citation missing for line ending with “idiopathic”.
- The introduction could be enriched by looking at the differences in research and reporting of problematic menstrual features between LMICs and HICs. I think this should be included to provide context. Also, there something to be said about perceptions – perhaps there is underreporting because there’s a lack of understanding or education around what is considered “problematic”

**Methods**

- REDCap (Research Electronic Data Capture) should appear first before being abbreviated. Also is there a citation available for this?
- For figure 2 – how was “started period” defined? Would one day of spotting suffice? Was three sequential months of bleeding? Girls often have irregular bleeding at menarche so it would be better to understand what “started period” actually meant to those reporting.
- The question around heavy bleeding is interesting as this is very subjective. Curious as to how this was interpreted by participants… Were they considering “heavy” in relation to their personal experiences or in comparison to their perception of what is “normal”?
- Similar comments for pain – how is a participant interpreting this question? It may have been better to look at the effect of pain on the ability to conduct usual daily activities.
- I commend the use of repeated measures.
- Further considerations should also explore the effect of medications/vaccinations on the menstrual cycle. There is a growing body of literature that suggests these could inform the menstrual cycle.

Are sufficient details of methods and materials provided to allow replication by others?

Partly

Is the rationale for creating the dataset(s) clearly described?

Yes

Are the datasets clearly presented in a useable and accessible format?

Yes

Are the protocols appropriate and is the work technically sound?

Yes

Reviewer Expertise:

menstrual health

I confirm that I have read this submission and believe that I have an appropriate level of expertise to confirm that it is of an acceptable scientific standard, however I have significant reservations, as outlined above.

# Response

**Published:** 2023-10-07

**Authors:** Gemma Sawyer

**Affiliations:** University of Bristol, Bristol, England, UK

*We thank the reviewers for their constructive and helpful comments. We have detailed our response below (in italics) and hope these address the reviewer’s critiques.*

Abstract  Overall, the abstract and key messages read very well. However, it would be helpful if the abstract noted where (geographical location) and when (time period including month and year) this research took place. *We have updated the abstract to include this information.*

*The variables presented in this paper have been collected in a UK, Bristol-based cohort since 1991. *  

Introduction  Very well written. However, please note the following edits: Citation missing for the first line. *T*

*he citations to support the text in the first line are in the following sentence which outlines the specific proportion ranges for each feature. *  

 Citation missing for line ending with “idiopathic”.

*Citations have been added. *  

 The introduction could be enriched by looking at the differences in research and reporting of problematic menstrual features between LMICs and HICs. I think this should be included to provide context. Also, there something to be said about perceptions – perhaps there is underreporting because there’s a lack of understanding or education around what is considered “problematic”.

*We have added a sentence to the first paragraph of the Introduction highlighting differences between LMICs and HICs.*  

Methods REDCap (Research Electronic Data Capture) should appear first before being abbreviated. Also is there a citation available for this?

*We have addressed the order of the abbreviation. The citation is included (reference 18; Harris et al. 2009).*  

 For figure 2 – how was “started period” defined? Would one day of spotting suffice? Was three sequential months of bleeding? Girls often have irregular bleeding at menarche so it would be better to understand what “started period” actually meant to those reporting.

*We have added a footnote to this figure to make it clear to the reader that ‘started period’ was based upon responses to the question “Have you started your period yet?” asked at each timepoint. No further information was provided in these questions to clarify how much bleeding would constitute having started their period.*  

 The question around heavy bleeding is interesting as this is very subjective. Curious as to how this was interpreted by participants… Were they considering “heavy” in relation to their personal experiences or in comparison to their perception of what is “normal”?

*We have added a few sentences to the final paragraph of the Further Considerations explaining the subjective nature of such questions and, whilst it may pose some limitations, it is also crucial to measure and understand people’s experiences of such symptoms. Subjective reporting of heavy bleeding is also used for diagnosis of heavy menstrual bleeding. *  

 Similar comments for pain – how is a participant interpreting this question? It may have been better to look at the effect of pain on the ability to conduct usual daily activities.

*See comment above regarding heavy bleeding – we have added a few sentences to address this. Whilst a useful way of measuring and understanding menstrual pain, these measures are not available from the ALSPAC cohort.*  

 I commend the use of repeated measures.   Further considerations should also explore the effect of medications/vaccinations on the menstrual cycle. There is a growing body of literature that suggests these could inform the menstrual cycle.

*We have added a sentence in the fourth paragraph of the Further Considerations highlighting that there are many factors we have not mentioned that are available in ALSPAC which researchers may want to consider, depending on their specific research question. We have not provided specific mention of medications/vaccinations as we do not aim to provide an exhaustive list of all such factors in this section.*
